# Low high-density lipoprotein and increased risk of several cancers: 2 population-based cohort studies including 116,728 individuals

**DOI:** 10.1186/s13045-020-00963-6

**Published:** 2020-09-30

**Authors:** Kasper Mønsted Pedersen, Yunus Çolak, Stig Egil Bojesen, Børge Grønne Nordestgaard

**Affiliations:** 1 Department of Clinical Biochemistry, Copenhagen University Hospital, Herlev and Gentofte Hospital, Borgmester Ib Juuls Vej 1, DK-2730 Herlev, Denmark; 2 The Copenhagen General Population Study, Copenhagen University Hospital, Herlev and Gentofte Hospital, Herlev, Denmark; 3 Faculty of Health and Medical Sciences, University of Copenhagen, Copenhagen, Denmark

**Keywords:** High-density lipoprotein, HDL cholesterol, Apolipoprotein A1, Cancer, Epidemiology

## Abstract

**Background:**

Increasing evidence suggests that high-density lipoprotein (HDL) may play a role in cancer development. We tested the hypothesis that low HDL levels are associated with increased risk of cancer.

**Methods:**

Individuals from two population-based cohorts, the Copenhagen General Population Study (2003–2015, *N* = 107 341), and the Copenhagen City Heart Study (1991–1994, *N* = 9387) were followed prospectively until end of 2016 to assess low plasma HDL cholesterol and apolipoprotein A1 as risk factors for cancer using Cox proportional hazard regression.

**Results:**

During up to 25 years follow-up, we observed 8748 cancers in the Copenhagen General Population Study and 2164 in the Copenhagen City Heart Study. In the Copenhagen General Population Study and compared to individuals with HDL cholesterol ≥ 2.0 mmol/L (≥ 77 mg/dL), multivariable adjusted hazard ratios (HRs) for any cancer were 1.13 (95% confidence interval 1.04–1.22) for individuals with HDL cholesterol of 1.5–1.99 mmol/L (58–77 mg/dL), 1.18 (1.08–1.30) for HDL cholesterol of 1.0–1.49 mmol/L (39–58 mg/dL), and 1.29 (1.12–1.48) for individuals with HDL cholesterol < 1.0 mmol/L (< 39 mg/dL). Correspondingly, compared to individuals with apolipoprotein A1 ≥ 190 mg/dL, HRs for any cancer were 1.06 (0.96–1.17) for individuals with apolipoprotein A1 of 160–189 mg/dL, 1.18 (1.07–1.30) for apolipoprotein A1 of 130–159 mg/dL, and 1.28 (1.13–1.46) for individuals with apolipoprotein A1 < 130 mg/dL. Among 27 cancer types, low HDL cholesterol and/or apolipoprotein A1 were associated with increased risk of multiple myeloma, myeloproliferative neoplasm, non-Hodgkin lymphoma, breast cancer, lung cancer, and nervous system cancer. Results were overall similar in women and men separately, and in the Copenhagen City Heart Study.

**Conclusions:**

Low HDL levels were associated with increased risk of several cancers. Increased risk was most pronounced for hematological and nervous system cancer, and to a minor extent for breast and respiratory cancer.

## Background

High-density lipoprotein (HDL) is the most abundant lipoprotein in the majority of species, implying that this particle has important functions in health and disease [1]. There is increasing evidence suggesting that HDL can regulate innate and adaptive immune responses and may have anti-oxidative, anti-apoptotic, and anti-inflammatory properties [2–6]. Through dysfunction of some of these properties, low HDL could play a role in the development of cancer [6].

Indeed, variation in HDL cholesterol level has been associated with risks of e.g. lung, endometrial, and colorectal cancer; however, results have been conflicting [6–18]. Further, it is unclear whether it is HDL cholesterol per se or apolipoprotein A1, the major protein component of HDL [6, 19], that best describe an association with risk of cancer.

We tested the hypothesis that low HDL levels are associated with increased risk of cancer. For this purpose, we used measured HDL cholesterol and apolipoprotein A1 in 116,728 individuals from two independent Danish population-based cohorts recruited during different time periods. These individuals were followed for up to 25 years for risk of any cancer and for 27 specific cancer types, using the national Danish Cancer Registry.

## Methods

### Study design and populations

The Copenhagen General Population Study is a population-based prospective cohort study recruited in 2003–2015 [20, 21]. All individuals in Denmark are assigned a unique identification number (Central Person Registration number) at birth or immigration and recorded in the national Danish Civil Registration System. Individuals aged ≥ 20 were selected and invited from the national Danish Civil Registration System to reflect the adult Danish general population. All participants completed a comprehensive questionnaire, underwent a physical examination, and gave blood for biochemical analyses. Questionnaires were reviewed in detail at the day of attendance by a healthcare professional together with the participant. In the present study, we included 107,341 individuals with information on measured HDL cholesterol and apolipoprotein A1.

The Copenhagen City Heart Study is a population-based prospective cohort study initiated in 1976–1978, with follow-up examinations in 1981–1983, 1991–1994, and 2001–2003, recruited and examined as the Copenhagen General Population Study but from different parts of Copenhagen [20, 21]. For independent confirmation, we included 9387 individuals from the 1991–1994 examination with information on measured HDL cholesterol and apolipoprotein A1.

Both studies were approved by institutional review boards and Danish ethical committees and conducted according to the Declaration of Helsinki (approval numbers: KF-V-100-2039/91 and H-KF-01-144/01). Written informed consent was obtained from all participants

### HDL cholesterol and apolipoprotein A1

HDL cholesterol and apolipoprotein A1 were measured using standard hospital assays immediately after collection of non-fasting blood samples [22]. HDL cholesterol was measured directly using a colorimetric assay, while apolipoprotein A1 was measured using an immunoturbidimetric assay (Konelab and Cobas). Assay precision was tested daily, while assay accuracy was tested monthly using an external quality control program.

### Cancer endpoints

Cancer endpoints were identified from the national Danish Cancer Registry, which was established in 1943 and records essentially all cancers diagnosed in Denmark [23]. Cancer diagnoses are reported by physicians and categorized based on location and histological examination by a fully trained pathologist using the World Health Organization criteria according to national Danish law. All cancer diagnoses (ICD-7: 140-205 and ICD-10: C00-D09) were included from baseline examination through December 2016; however, non-melanoma skin cancers were excluded, as these are very frequent and likely have a distinct etiology from other cancer forms. Information on myeloproliferative neoplasm was obtained from the national Danish Patient Registry, as done previously [24, 25]. By using the unique Central Person Registration number provided to everyone in Denmark at birth or immigration and linking it with the national Danish Cancer Registry and the national Danish Patient Registry, no person was lost to follow-up, and individuals who emigrated were censored at the date of emigration (*N* = 352 for the Copenhagen General Population Study and *N* = 66 for the Copenhagen City Heart Study).

### Covariates

Date of birth and sex was obtained from the national Danish Civil Registration System [26]. Body mass index was calculated as measured weight divided by measured height squared (kg/m^2^). Smoking status was defined as never, former, or current smoker. Cumulative tobacco consumption was calculated in pack-years based on information on age at smoking initiation and cessation (or for current smokers until age at baseline examination), duration of tobacco consumption, and amount of consumed tobacco (number of daily consumed cigarettes, cheroots, and cigars and grams of weekly consumed pipe tobacco), i.e., a pack-year was 20 cigarettes or equivalent smoked daily for a year. Alcohol consumption was reported in units per week (1 unit = 12 g of alcohol). Leisure-time physical activity was reported according to hours per week and degree of activity. Education was based on years attending school. Income was reported as annual household income. Plasma triglycerides and C-reactive protein were measured using standard hospital assays; the latter using a high-sensitive assay. Lipid-lowering therapy was self-reported and mainly statins. Information on ischemic heart disease (ICD-8: 410-414 and ICD-10: I20-I25) and chronic obstructive pulmonary disease (ICD-8: 491-492 and ICD-10: J41-J44) was obtained from the national Danish Patient Registry. Information on diabetes was based on self-report, non-fasting plasma glucose > 11 mmol/L, use of anti-diabetic medication, and/or the national Danish Patient Registry (ICD-8: 249-250 and ICD-10: E10-E14).

### Statistical analyses

Analyses were carried out using STATA/SE 13.1 (StataCorp. LP, StataCorp, College Station, TX), and 2-sided *P* values < 0.05 were considered statistically significant. Baseline characteristics were compared using Pearson’s chi-squared or Kruskal-Wallis test. Association of HDL cholesterol and apolipoprotein A1 with risk of cancer was investigated using Cox proportional hazard regression. We used age as the underlying timescale (=age adjusted) with left truncation (=delayed entry) at study examination. Individuals with event before the baseline examination were excluded from the particular analysis, which is why the total number of individuals varies across cancer types. Some individuals had more than one cancer, which is why the sum of individual first cancers exceeds the number of any first cancer. Only the appropriate sex was included in the analyses of cancers in breast, corpus uteri, testis, prostate, ovary, and cervix uteri. Restricted cubic spline models were used to assess non-linear relationships with three knots placed according to Harrell’s recommended percentiles [27], and the median values of HDL cholesterol and apolipoprotein A1 were chosen as reference. Associations were also investigated in a setting with all-cause mortality and emigration as competing events using the methods proposed by Fine and Gray [28]. Wald’s test was used to assess potential effect modification (=interaction). As sensitivity analyses, we excluded individuals with events within 1–3 years after the baseline examination to investigate potential reverse causation. Analyses were multivariable adjusted for potential confounders, that is, age (as timescale), sex (except for analyses of cancers in breast, corpus uteri, testis, prostate, ovary, and cervix uteri in which only the appropriate sex was included), body mass index, smoking status, cumulative tobacco consumption, alcohol intake, leisure-time physical activity, education, income, plasma triglycerides, lipid-lowering therapy, C-reactive protein, and baseline chronic disease (ischemic heart disease, chronic obstructive pulmonary disease, and diabetes); plasma triglycerides are inversely associated with HDL cholesterol levels [29]. Since information on covariates was > 99% complete, missing values were imputed with multiple imputation using chained equations [30]; however, results were similar using complete case analysis. All risk estimates and confidence intervals were corrected for regression dilution bias with a nonparametric method, as done previously [20, 21, 31].

## Results

Among individuals from the Copenhagen General Population Study, 8748 (8.1%) were diagnosed with cancer during up to 13 years follow-up (median 7.4 years). Among individuals from the Copenhagen City Heart Study, 2164 (23%) were diagnosed with cancer during up to 25 years follow-up (median 18 years). Baseline characteristics are presented in the supplement (Tables S1–S4).

### Any cancer

**Fig. 1:**
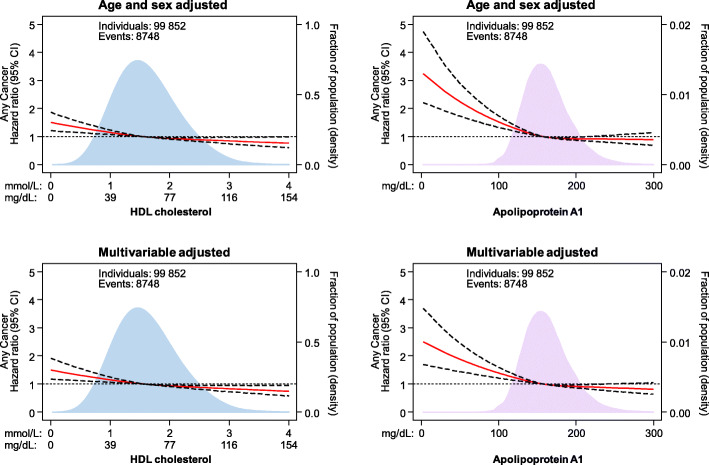
Association of HDL cholesterol and apolipoprotein A1 levels with risk of any cancer in individuals from the Copenhagen General Population Study. Hazard ratios and 95% confidence intervals (CIs) were obtained from Cox proportional hazards regression with restricted cubic splines. Multivariable adjustment included age, sex, body mass index, smoking status, cumulative tobacco consumption, alcohol intake, leisure-time physical activity, education, income, plasma triglycerides, lipid-lowering therapy, C-reactive protein, and baseline chronic disease (ischemic heart disease, chronic obstructive pulmonary disease, and diabetes). The median values of HDL cholesterol and apolipoprotein A1 were chosen as reference. The red line represents the hazard ratio and the dotted lines 95% CIs. Areas of light blue and purple represent the distribution of levels of HDL cholesterol and apolipoprotein A1, respectively. HDL=high-density lipoprotein

**Fig. 2:**
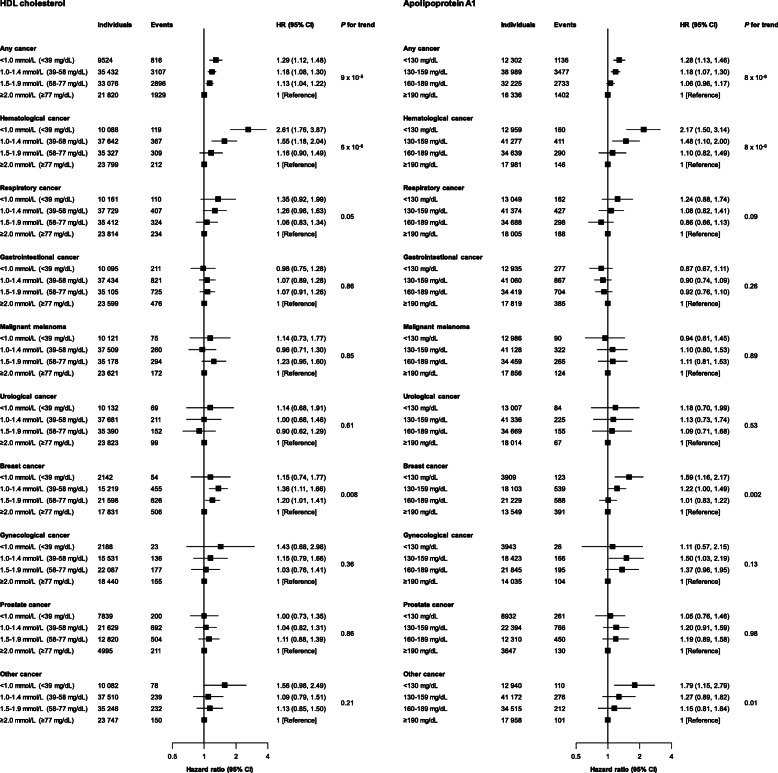
Association of HDL cholesterol and apolipoprotein A1 levels with risk of any cancer and nine major cancer forms in individuals from the Copenhagen General Population Study. Hazard ratios and 95% confidence intervals (CIs) were obtained from Cox proportional hazards regression multivariable adjusted for age, sex, body mass index, smoking status, cumulative tobacco consumption, alcohol intake, leisure-time physical activity, education, income, plasma triglycerides, lipid-lowering therapy, C-reactive protein, and baseline chronic disease (ischemic heart disease, chronic obstructive pulmonary disease, and diabetes). *P* for trend was obtained from Wald’s test. Numbers vary slightly due to exclusion of individuals with baseline cancer relevant for the specific cancer form. The sum of the nine major cancer forms exceeds the number of any cancer, as some individuals developed more than one specific cancer form. Hematological cancer included: non-Hodgkin lymphoma, Hodgkin’s lymphoma, multiple myeloma, leukemia, and myeloproliferative neoplasm. Respiratory cancer included: larynx and lung. Gynecological cancer included: cervix uteri, corpus uteri, and ovaries. Urological cancer included: kidney, bladder, and excretory urinary tract. Gastrointestinal cancer included: oral cavity and pharynx, esophagus, stomach, colon/rectum/anus, liver and biliary tract, and pancreas. HDL=high-density lipoprotein

Low levels of HDL cholesterol and apolipoprotein A1 were associated with increased risk of any cancer in the Copenhagen General Population Study (Figs. 1 and 2). Compared to individuals with HDL cholesterol ≥ 2.0 mmol/L (≥ 77 mg/dL), multivariable adjusted hazard ratio (HR) for any cancer was 1.13 (95% confidence interval [CI] 1.04–1.22) for individuals with HDL cholesterol of 1.5–1.99 mmol/L (58–77 mg/dL), 1.18 (1.08–1.30) for individuals with HDL cholesterol of 1.0–1.49 mmol/L (39–58 mg/dL), and 1.29 (1.12–1.48) for individuals with HDL cholesterol < 1.0 mmol/L (< 39 mg/dL) (*P* for trend = 9 × 10^−5^; Fig. 2, upper left panel). Correspondingly, compared to individuals with apolipoprotein A1 ≥ 190 mg/dL, multivariable adjusted HR for any cancer was 1.06 (0.96–1.17) for individuals with apolipoprotein A1 of 160–189 mg/dL, 1.18 (1.07–1.30) for individuals with apolipoprotein A1 of 130–159 mg/dL, and 1.28 (1.13–1.46) for individuals with apolipoprotein A1 < 130 mg/dL (*P* for trend = 8 × 10^−6^; Fig. 2, upper right panel).

### Specific cancer types

**Fig. 3:**
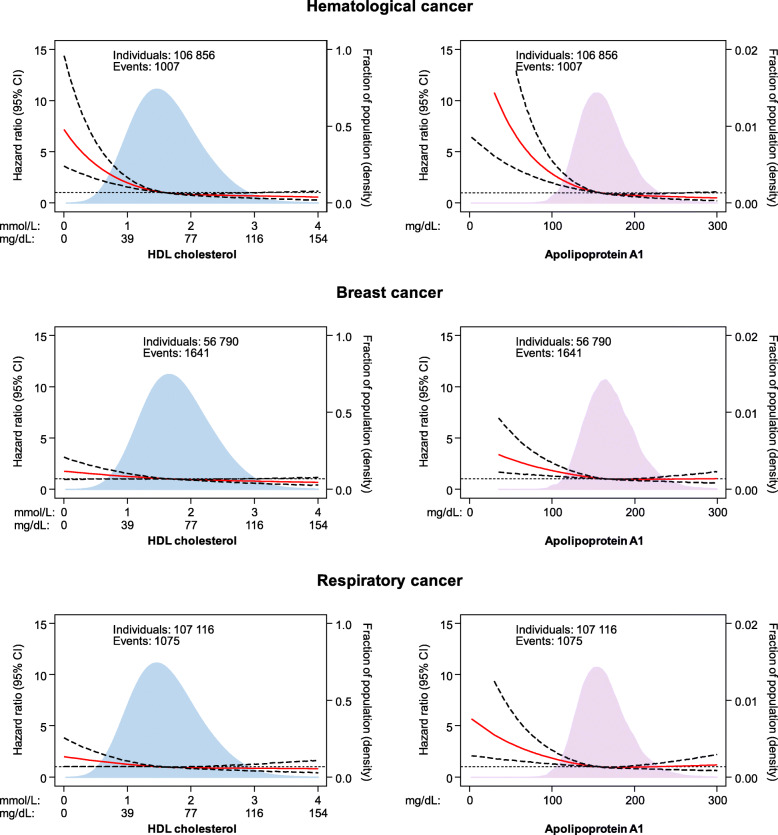
Association of HDL cholesterol and apolipoprotein A1 levels with risk of hematological, breast, and respiratory cancer in individuals from the Copenhagen General Population Study. Hazard ratios and 95% confidence intervals (CIs) were obtained from Cox proportional hazards regression with restricted cubic splines multivariable adjusted for age, sex, body mass index, smoking status, cumulative tobacco consumption, alcohol intake, leisure-time physical activity, education, income, plasma triglycerides, lipid-lowering therapy, C-reactive protein, and baseline chronic disease (ischemic heart disease, chronic obstructive pulmonary disease, and diabetes). The median values of HDL cholesterol and apolipoprotein A1 were chosen as reference. The red line represents the hazard ratio and the dotted lines 95% CIs. Areas of light blue and purple represent the distribution of levels of HDL cholesterol and apolipoprotein A1, respectively. Numbers vary slightly due to exclusion of individuals with baseline cancer relevant for the specific cancer form. Hematological cancer included: non-Hodgkin lymphoma, Hodgkin’s lymphoma, multiple myeloma, leukemia, and myeloproliferative neoplasm. Respiratory cancer included: larynx and lung. HDL=high-density lipoprotein

Among nine major cancer forms, low levels of HDL cholesterol and apolipoprotein A1 were associated with increased risk of hematological cancer, breast cancer, and respiratory cancer, but not with gynecological cancer, urological cancer, gastrointestinal cancer, prostate cancer, malignant melanoma, or other cancer in the Copenhagen General Population Study (Figs. 2 and 3 and Figures S1 and S2).

**Fig. 4:**
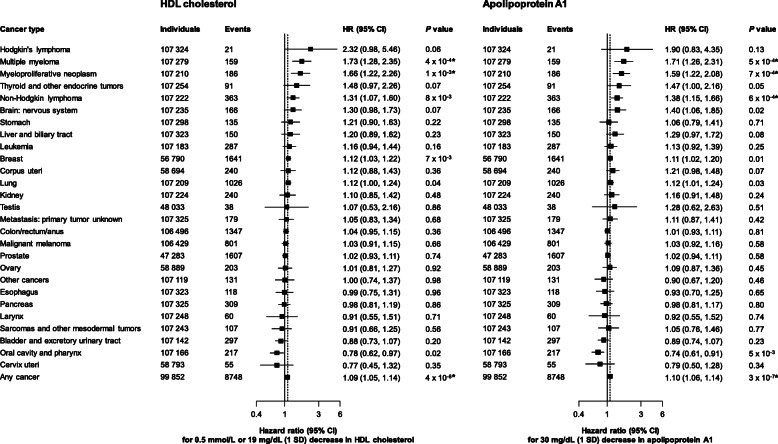
Risk of 27 specific cancer types in the Copenhagen General Population Study according to HDL cholesterol and apolipoprotein A1 levels. Hazard ratios (HRs) and 95% confidence intervals (CIs) were obtained from Cox proportional hazards regression multivariable adjusted for age, sex, body mass index, smoking status, cumulative tobacco consumption, alcohol intake, leisure-time physical activity, education, income, plasma triglycerides, lipid-lowering therapy, C-reactive protein, and baseline chronic disease (ischemic heart disease, chronic obstructive pulmonary disease, and diabetes). Numbers vary slightly due to exclusion of individuals with baseline cancer for the relevant type of cancer. The sum of the 27 specific cancer types exceeds the number of any cancer, as some participants developed more than one type of cancer. HDL=high-density lipoprotein. *P value <0.05 after adjustment for 28 multiple comparisons according to the method of Bonferroni; *P* value=0.05 is equivalent to *P*=0.05/28=0.002

Among 27 specific cancer types, low levels of HDL cholesterol and/or apolipoprotein A1 were associated with increased risk of multiple myeloma, myeloproliferative neoplasm, non-Hodgkin lymphoma, breast cancer, lung cancer, and nervous system cancer, and with reduced risk of oral cavity and pharynx cancer (Fig. 4 and Figures S3 and S4). When taking multiple comparisons into account, only risk of multiple myeloma, myeloproliferative neoplasm, and non-Hodgkin lymphoma had *P* values < 0.05 (Fig. 4).

Local tumor expression levels of apolipoprotein A1 might influence inflammatory pathways, which in turn might impact growth of neoplastic cells. Indeed, for lung adenocarcinoma and squamous cell carcinoma, expression levels of both apolipoprotein A1 RNA and protein in the Cancer Genome Atlas Program (GEPIA interactive web server) and the Clinical Proteomic Tumor Analysis Consortium (UALCAN interactive web server) were significantly decreased compared to in normal tissue [32–34]. For invasive breast carcinoma, apolipoprotein A1 RNA expression did not seem to differ, whereas protein expression was decreased in tumor tissue. No broad classification for hematological malignancies were available in the databases.

### Independent confirmation

**Fig. 5:**
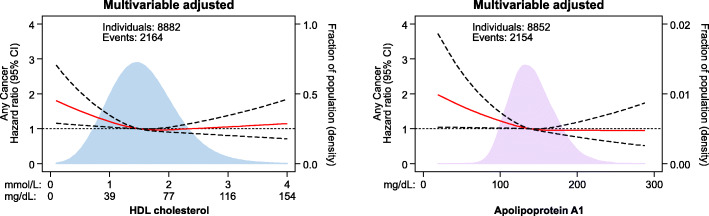
Association of HDL cholesterol and apolipoprotein A1 levels with risk of any cancer in individuals from the Copenhagen City Heart Study. Hazard ratios and 95% confidence intervals (CIs) were obtained from Cox proportional hazards regression with restricted cubic splines multivariable adjusted for age, sex, body mass index, smoking status, cumulative tobacco consumption, alcohol intake, leisure-time physical activity, education, income, plasma triglycerides, lipid-lowering therapy, C-reactive protein, and baseline chronic disease (ischemic heart disease, chronic obstructive pulmonary disease, and diabetes). The median values of HDL cholesterol and apolipoprotein A1 were chosen as reference. The red line represents the hazard ratio and the dotted lines 95% CIs. Areas of light blue and purple represent the distribution of levels of HDL cholesterol and apolipoprotein A1, respectively. Number of individuals and events vary slightly due to lacking information on apolipoprotein A1 in some individuals. HDL=high-density lipoprotein

Low levels of HDL cholesterol and apolipoprotein A1 were also associated with increased risk of any cancer in the Copenhagen City Heart Study with comparable risk estimates to those observed in the Copenhagen General Population Study (compare Fig. 1 to Fig. 5). On direct comparison for HDL cholesterol above 2 mmol/L (77 mg/dL), the risk of any cancer was nominally higher in the Copenhagen City Heart Study but not in the Copenhagen General Population Study; however, the 95% CI for HRs in the two studies overlapped, likely suggesting similar results.

For hematological and breast cancer, we observed similar results in the Copenhagen City Heart Study when compared to the Copenhagen General Population Study (compare Fig. 3 with Figure S5). In contrast, neither HDL cholesterol nor apolipoprotein A1 were associated with risk of respiratory cancer in individuals from the Copenhagen City Heart Study (Figure S5). Common for these analyses were wide confidence intervals due to limited statistical power. Similarly, due to a smaller sample size, risk of 27 specific cancer types could not be investigated in the Copenhagen City Heart Study with sufficient statistical power.

### Sensitivity and subgroup analyses

Results were similar for HDL cholesterol and apolipoprotein A1 in men and women separately (compare Figs. 1 and 3 and Figure S1 with Figures S6, S7, and S8; results for apolipoprotein A1 and major cancer forms are not shown). Results for any cancer were similar when stratified according to covariates, without evidence of interaction after taking multiple comparisons into account (Figure S9). Results for any cancer and the nine major cancer forms were also similar in a setting with all-cause mortality and emigration as competing events (compare Fig. 2 with Figure S10). Further, results for any, hematological, breast, and respiratory cancer were similar when individuals who developed cancer within 1–3 years after baseline examination were excluded, with the aim of reducing potential reverse causation (Figures S11–S13). Interestingly, when HDL cholesterol and apolipoprotein A1 were included in the same analyses, we found that risk estimates for HDL cholesterol overlapped with 1, while risk estimates for apolipoprotein A1 did not (Figure S14).

## Discussion

In two independent Danish cohorts totaling 116,728 individuals and up to 25 years of follow-up, we found that low levels of HDL cholesterol and apolipoprotein A1 were associated with increased risk of several cancers. Increased risk was most pronounced for hematological and nervous system cancer, and to a minor extent for breast and respiratory cancer. Importantly, chance findings due to multiple comparison needs to be considered when interpreting our results on specific cancer types.

Mechanisms behind the present findings may be related to the ability of HDL to regulate proliferative and inflammatory pathways in cancer development through its proposed immunomodulatory, anti-oxidative, anti-apoptotic, and anti-inflammatory properties [2–6, 21, 35, 36]. By interacting with lipid rafts on cellular membranes enriched in immune cell receptors [37], HDL could modulate and prime the immune system towards a more beneficial anti-cancer state. Another interesting aspect is that HDL cholesterol and apolipoprotein A1 have been shown to inhibit proliferation of hematopoietic stem and progenitor cells [3, 38, 39]. The fact that low HDL cholesterol and apolipoprotein A1 were associated with increased risk of hematological cancers suggests that HDL particles may be essential in the tight control of proliferation and homeostasis of the hematopoietic system, perhaps hindering malignant transformation. The interplay between HDL cholesterol and apolipoprotein A1 with receptors responsible for cholesterol transport such as ATP-binding cassette transporters and scavenger receptors in the development of hematological cancers could be explored in future studies. Whether this potential mechanism is also involved in development of non-hematological cancer forms can only be speculated on.

Other mechanistic studies suggest that apolipoprotein A1 itself has anti-tumorigenic properties through reduced angiogenesis, modification of immune cells, and enhancement of cholesterol efflux and reverse cholesterol transport from cancer cells; mechanisms that could potentially inhibit proliferation or growth of tumor cells [40, 41]. An important role of apolipoprotein A1 can also be supported by our last analysis, where apolipoprotein A1 was still associated with risk of cancer independent from HDL cholesterol, while the opposite was not applicable (Figure S14). Thus, a potential protective role of HDL cholesterol may be mediated through the number of HDL particles and its components such as apolipoprotein A1 rather than by the cholesterol level itself.

Alternatively, results could also merely be reflective of an epiphenomenon of cancer-related inflammation and cancer cell renewal, e.g., through cancer cell up-regulation of HDL cholesterol transporting scavenger receptors [6, 42, 43]. This alternative explanation suggests bias in form of reverse causation. We tried to address this issue by excluding individuals developing cancer within the first 3 years of follow-up, which yielded similar results and therefore suggests absence or minor influence of reverse causation. However, in some cancer types such as myeloproliferative neoplasm, it can take several years before the cancers become overt and consequently diagnosed. Thus, we cannot completely exclude that low HDL cholesterol and apolipoprotein A1 may just represent preclinical indicators of early cancer development.

Previous studies on HDL cholesterol and cancer have recently been summarized in a review article [6]. In the Women’s Health Study, an inverse association of HDL cholesterol and/or apolipoprotein A1 with any cancer, colorectal cancer, and lung cancer was observed [7]. Meta-analyses of randomized controlled trials have showed that low levels of HDL cholesterol were associated with increased risk of any cancer as well [9]. Correspondingly, cohort studies and meta-analyses thereof have shown similar inverse associations [6, 10–12, 44]. However, other similar studies were unable to observe an association between HDL cholesterol and risk of cancer [6, 13, 18, 45]. A meta-analysis of Mendelian randomization studies has shown an association between the *APOE* ε2/ε3/ε4 polymorphism and risk of cancer in Asians [8]. However, since *APOE* is highly pleiotropic, the association could be driven by other lipid fractions than HDL cholesterol. In contrast, other Mendelian randomization studies have failed to show a causal association of HDL cholesterol and risk of colorectal, prostate, lung, and breast cancer [14, 16, 17, 46], and in one of the studies, genetically high levels of HDL cholesterol was contrarily associated with increased risk of estrogen receptor-positive breast cancer [16]. In the present study by using two independent population-based cohorts, we investigated the association of low HDL levels with risk of any cancer and 27 specific cancer types. We observed that low levels of HDL cholesterol and apolipoprotein A1 were associated with an increased risk of several cancers, with the increased risk being most pronounced for hematological and nervous system cancer, and to a minor extent for breast and respiratory cancer.

Strengths of the present study include the large number of randomly selected individuals, detailed information on important covariates, long follow-up time without any losses to follow-up, and the use of the national Danish Cancer Registry to identify cancer endpoints, which has shown high completeness and validity [23]. Furthermore, overall results were similar in two independent population-based cohorts with individuals recruited in two different time-periods.

A limitation is the study design which cannot be used to infer causality. Despite observing similar results after multivariable adjustment and after excluding those diagnosed with cancer up to 3 years after the baseline examination, we cannot exclude residual confounding and/or reverse causation. Another limitation is that we only had information on HDL cholesterol and apolipoprotein A1 at the baseline examination and not during follow-up. Lastly, we studied white individuals only.

## Conclusions

Low HDL cholesterol and apolipoprotein A1 were associated with increased risk of several cancers. Increased risk was most pronounced for hematological and nervous system cancer, and to a minor extent for breast and respiratory cancer. Further investigations are needed to identify which HDL component(s) and/or HDL subpopulation(s) are responsible for the observed associations.

## Supplementary information

**Additional file 1: Table S1.** Baseline characteristics of individuals in the Copenhagen General Population Study according to HDL cholesterol levels. **Table S2.** Baseline characteristics of individuals in the Copenhagen General Population Study according to apolipoprotein A1 levels. **Table S3.** Baseline characteristics of individuals in the Copenhagen City Heart Study according to HDL cholesterol levels. **Table S4.** Baseline characteristics of individuals in the Copenhagen City Heart Study according to apolipoprotein A1 levels. **Figure S1.** Association of HDL cholesterol levels with risk of gynecological, urological, gastrointestinal, prostate, malignant melanoma, and other cancer in individuals from the Copenhagen General Population Study. **Figure S2.** Association of apolipoprotein A1 levels with risk of gynecological, urological, gastrointestinal, prostate, malignant melanoma, and other cancer in individuals from the Copenhagen General Population Study. **Figure S3a and S3b.** Association of HDL cholesterol levels with risk of 27 specific cancer types in individuals from the Copenhagen General Population Study. **Figure S4a and S4b.** Association of apolipoprotein A1 levels with risk of 27 specific cancer types in individuals from the Copenhagen General Population Study. **Figure S5.** Association of HDL cholesterol and apolipoprotein A1 levels with risk of hematological, breast, and respiratory cancer in individuals from the Copenhagen City Heart Study. **Figure S6.** Association of HDL cholesterol and apolipoprotein A1 levels with risk of any cancer in individuals from the Copenhagen General Population Study according to sex. **Figure S7.** Association of HDL cholesterol levels with risk of eight major cancer forms in women from the Copenhagen General Population Study. **Figure S8.** Association of HDL cholesterol levels with risk of seven major cancer forms in men from the Copenhagen General Population Study. **Figure S9.** Association of low versus high HDL cholesterol levels with risk of any cancer in individuals from the Copenhagen General Population Study according to covariates. **Figure S10.** Association of HDL cholesterol and apolipoprotein A1 levels with risk of any cancer and nine major cancer forms in individuals from the Copenhagen General Population Study with all-cause mortality and emigration as competing events. **Figure S11.** Association of HDL cholesterol and apolipoprotein A1 levels with risk of any cancer in individuals from the Copenhagen General Population Study excluding individuals with 1, 2, and 3 years of follow-up. **Figure S12.** Association of HDL cholesterol levels with risk of hematological, breast, and respiratory cancer in individuals from the Copenhagen General Population Study excluding individuals with 1, 2, and 3 years of follow-up. **Figure S13.** Association of apolipoprotein A1 levels with risk of hematological, breast, and respiratory cancer in individuals from the Copenhagen General Population Study excluding individuals with 1, 2, and 3 years of follow-up. **Figure S14.** HDL cholesterol and apolipoprotein A1 levels adjusted risk of any cancer in individuals from the Copenhagen General Population Study.

## Data Availability

Data that further support the findings of this study are available from the corresponding author upon reasonable request.

## Abbreviations

CI: Confidence interval
HDL: High-density lipoprotein
HR: Hazard ratio
