# Supplementary material for: Low high-density lipoprotein and increased risk of several cancers: 2 population-based cohort studies including 116,728 individuals
Source: J Hematol Oncol. 2020 Sep 30;13:129. doi: 10.1186/s13045-020-00963-6 (PMC7528381; doi:10.1186/s13045-020-00963-6)
Supplement: Supplementary file 1 — Additional file 1: Table S1. Baseline characteristics of individuals in the Copenhagen General Population Study according to HDL cholesterol levels. Table S2. Baseline characteristics of individuals in the Copenhagen General Population Study according to apolipoprotein A1 levels. Table S3. Baseline characteristics of individuals in the Copenhagen City Heart Study according to HDL cholesterol levels. Table S4. Baseline characteristics of individuals in the Copenhagen City Heart Study according to apolipoprotein A1 levels. Figure S1. Association of HDL cholesterol levels with risk of gynecological, urological, gastrointestinal, prostate, malignant melanoma, and other cancer in individuals from the Copenhagen General Population Study. Figure S2. Association of apolipoprotein A1 levels with risk of gynecological, urological, gastrointestinal, prostate, malignant melanoma, and other cancer in individuals from the Copenhagen General Population Study. Figure S3a and S3b. Association of HDL cholesterol levels with risk of 27 specific cancer types in individuals from the Copenhagen General Population Study. Figure S4a and S4b. Association of apolipoprotein A1 levels with risk of 27 specific cancer types in individuals from the Copenhagen General Population Study. Figure S5. Association of HDL cholesterol and apolipoprotein A1 levels with risk of hematological, breast, and respiratory cancer in individuals from the Copenhagen City Heart Study. Figure S6. Association of HDL cholesterol and apolipoprotein A1 levels with risk of any cancer in individuals from the Copenhagen General Population Study according to sex. Figure S7. Association of HDL cholesterol levels with risk of eight major cancer forms in women from the Copenhagen General Population Study. Figure S8. Association of HDL cholesterol levels with risk of seven major cancer forms in men from the Copenhagen General Population Study. Figure S9. Association of low versus high HDL cholesterol levels with risk of any cance [file 13045_2020_963_MOESM1_ESM.docx]

**Supplementary material**

**Low high-density lipoprotein and increased risk of several cancers: two population-based cohort studies including 116 728 individuals**

Kasper Mønsted Pedersen, MD; Yunus Çolak, MD, PhD; Stig Egil Bojesen, MD, DMSc; and Børge Grønne Nordestgaard, MD, DMS

**Table S1.** Baseline characteristics of individuals in the Copenhagen General Population Study according to HDL cholesterol levels.
**Table S2.** Baseline characteristics of individuals in the Copenhagen General Population Study according to apolipoprotein A1 levels.

**Table S3.** Baseline characteristics of individuals in the Copenhagen City Heart Study according to HDL cholesterol levels.
**Table S4.** Baseline characteristics of individuals in the Copenhagen City Heart Study according to apolipoprotein A1 levels.
**Figure S1.** Association of HDL cholesterol levels with risk of gynecological, urological, gastrointestinal, prostate, malignant melanoma, and other cancers in individuals from the Copenhagen General Population Study.
**Figure S2.** Association of apolipoprotein A1 levels with risk of gynecological, urological, gastrointestinal, prostate, malignant melanoma, and other cancers in individuals from the Copenhagen General Population Study.
**Figure S3a and S3b.** Association of HDL cholesterol levels with risk of 27 specific cancer types in individuals from the Copenhagen General Population Study.
**Figure S4a and S4b.** Association of apolipoprotein A1 levels with risk of 27 specific cancer types in individuals from the Copenhagen General Population Study.
**Figure 5.** Association of HDL cholesterol and apolipoprotein A1 levels with risk of hematological, breast, and respiratory cancers in individuals from the Copenhagen City Heart Study.
**Figure S6.** Association of HDL cholesterol and apolipoprotein A1 levels with risk of any cancer in individuals from the Copenhagen General Population Study according to sex.
**Figure S7.** Association of HDL cholesterol levels with risk of eight major cancer forms in women from the Copenhagen General Population Study.
**Figure S8.** Association of HDL cholesterol levels with risk of seven major cancer forms in men from the Copenhagen General Population Study.
**Figure S9.** Association of low versus high HDL cholesterol levels with risk of any cancer in individuals from the Copenhagen General Population Study according to covariates.
**Figure S10.** Association of HDL cholesterol and apolipoprotein A1 levels with risk of any cancer and nine major cancer forms in individuals from the Copenhagen General Population Study with all-cause mortality and emigration as competing events.
**Figure S11.** Association of HDL cholesterol and apolipoprotein A1 levels with risk of any cancer in individuals from the Copenhagen General Population Study excluding individuals with 1, 2, and 3 years of follow-up.
**Figure S12.** Association of HDL cholesterol levels with risk of hematological, breast, and respiratory cancers in individuals from the Copenhagen General Population Study excluding individuals with 1, 2, and 3 years of follow-up.
**Figure S13.** Association of apolipoprotein A1 levels with risk of hematological, breast, and respiratory cancers in individuals from the Copenhagen General Population Study excluding individuals with 1, 2, and 3 years of follow-up.
**Figure S14.** HDL cholesterol and apolipoprotein A1 levels adjusted risk of any cancer in individuals from the Copenhagen General Population Study.

**Table S1.** Baseline characteristics of individuals in the Copenhagen General Population Study according to HDL cholesterol levels.

|  | **HDL cholesterol, mmol/L (mg/dL)** | | | |  |
| --- | --- | --- | --- | --- | --- |
|  | **<1.0 (<39)**  **n=10 190** | **1.0-1.49 (39-58)**  **n=37 804** | **1.5-1.99 (58-77)**  **n=35 473** | **≥2.0 (≥77)**  **n=23 874** | ***P* value** |
| Age, years | 55 (46-65) | 56 (47-66) | 58 (48-68) | 61 (52-69) | <0.001 |
| Men, no. (%) | 7972 (78) | 22 045 (58) | 13 103 (37) | 5151 (22) | <0.001 |
| Body mass index, kg/m^2^ | 28.3 (25.8-31.3) | 26.6 (24.3-29.4) | 25.0 (22.8-27.5) | 23.8 (21.9-26.1) | <0.001 |
| Current smokers, no. (%) | 2501 (25) | 7346 (19) | 5383 (15) | 3210 (13) | <0.001 |
| Cumulative tobacco consumption, pack-years^a^ | 21 (10-35) | 18 (8-32) | 15 (5-29) | 13 (5-26) | <0.001 |
| Alcohol consumption, units/week^b^ | 7 (3-13) | 7 (3-14) | 8 (4-15) | 10 (5-16) | <0.001 |
| Physical inactivity, no. (%)^c^ | 1135 (11) | 2824 (7.5) | 1742 (5.0) | 914 (3.9) | <0.001 |
| Low education, no. (%)^d^ | 1623 (16) | 6073 (16) | 5386 (15) | 3780 (16) | 0.009 |
| Low annual household income, no. (%)^e^ | 1259 (12) | 4637 (12) | 4326 (12) | 3182 (14) | <0.001 |
| Lipid-lowering therapy, no. (%) | 1516 (15) | 4845 (13) | 3972 (11) | 2570 (11) | <0.001 |
| High-sensitive C-reactive protein, mg/dL | 1.8 (1.2-3.2) | 1.5 (1.0-2.5) | 1.3 (0.9-2.1) | 1.2 (0.8-1.8) | <0.001 |
| Any prevalent chronic disease, no. (%) | 1709 (17) | 4634 (12) | 3203 (9.0) | 2064 (8.7) | <0.001 |
| Ischemic heart disease, no. (%) | 861 (8.5) | 2557 (6.8) | 1739 (4.9) | 1016 (4.3) | <0.001 |
| Diabetes, no. (%) | 925 (9.1) | 1880 (5.0) | 1103 (3.1) | 639 (2.7) | <0.001 |
| Chronic obstructive pulmonary disease, no. (%) | 204 (2.0) | 777 (2.1) | 693 (2.0) | 586 (2.5) | <0.001 |
| HDL cholesterol |  |  |  |  |  |
| mmol/L | 0.9 (0.8-0.9) | 1.3 (1.2-1.4) | 1.7 (1.6-1.8) | 2.3 (2.1-2.5) | <0.001 |
| mg/dL | 34 (30–36) | 49 (45–54) | 66 (62–71) | 88 (82–98) | <0.001 |
| Apolipoprotein A1, mg/dL | 128 (117-140) | 145 (133-157) | 165 (153-177) | 194 (179-212) | <0.001 |
| Triglycerides |  |  |  |  |  |
| mmol/L | 2.6 (1.8-3.7) | 1.7 (1.2-2.4) | 1.2 (0.9-1.7) | 1.0 (0.8-1.3) | <0.001 |
| mg/dL | 231 (162–328) | 151 (107–213) | 109 (81–150) | 86 (66–116) | <0.001 |

Data is summarized as medians (IQR), or numbers (%). *P* values were obtained from the Pearson’s chi-square or Kruskal–Wallis test. HDL=high-density lipoprotein.
^a^Included only former and current smokers.
^b^12 grams=1 unit of alcohol.
^c^Being completely sedentary or doing light physical activity less than 2 hours/week in leisure-time.
^d^<9 years of school attendance.
^e^Annual household income <200 000 DKK (~30 000 USD).

**Table S2.** Baseline characteristics of individuals in the Copenhagen General Population Study according to apolipoprotein A1 levels.

|  | **Apolipoproteinn A1, mg/dL** | | | |  |
| --- | --- | --- | --- | --- | --- |
|  | **<130**  **n=13 071** | **130-159**  **n=41 458** | **160-189**  **n=34 757** | **≥190**  **n=18 055** | ***P* value** |
| Age, years | 53 (44-65) | 56 (46-66) | 59 (50-68) | 62 (54-70) | <0.001 |
| Men, no. (%) | 9087 (70) | 22 812 (55) | 12 613 (36) | 3759 (21) | <0.001 |
| Body mass index, kg/m^2^ | 26.6 (24.1-29.6) | 26.0 (23.6-28.9) | 25.3 (22.9-28.0) | 24.5 (22.3-27.1) | <0.001 |
| Current smokers, no. (%) | 2986 (23) | 7600 (18) | 5186 (15) | 2668 (15) | <0.001 |
| Cumulative tobacco consumption, pack-years^a^ | 19 (8-34) | 17 (7-30) | 15 (5-29) | 15 (5-30) | <0.001 |
| Alcohol consumption, units/week^b^ | 6 (2-11) | 7 (3-14) | 9 (4-16) | 11 (6-18) | <0.001 |
| Physical inactivity, no. (%)^c^ | 1211 (9.4) | 2784 (6.8) | 1831 (5.3) | 789 (4.4) | <0.001 |
| Low education, no. (%)^d^ | 2078 (16) | 6459 (16) | 5412 (16) | 2913 (16) | 0.28 |
| Low annual household income, no. (%)^e^ | 1738 (13) | 4957 (12) | 4218 (12) | 2491 (14) | <0.001 |
| Lipid-lowering therapy, no. (%) | 1675 (13) | 4763 (12) | 4074 (12) | 2391 (13) | <0.001 |
| High-sensitive C-reactive protein, mg/dL | 1.5 (1.0-2.9) | 1.4 (0.9-2.3) | 1.4 (0.9-2.2) | 1.4 (0.9-2.1) | <0.001 |
| Any prevalent chronic disease, no. (%) | 2013 (15) | 4609 (11) | 3346 (10) | 1642 (9.1) | <0.001 |
| Ischemic heart disease, no. (%) | 1181 (9.0) | 2497 (6.0) | 1727 (5.0) | 768 (4.3) | <0.001 |
| Diabetes, no. (%) | 914 (7.0) | 1867 (4.5) | 1224 (3.5) | 542 (3.0) | <0.001 |
| Chronic obstructive pulmonary disease, no. (%) | 266 (2.0) | 790 (1.9) | 733 (2.1) | 471 (2.6) | <0.001 |
| HDL cholesterol |  |  |  |  |  |
| mmol/L | 1.0 (0.9-1.2) | 1.4 (1.2-1.6) | 1.8 (1.5-2.0) | 2.3 (2.0-2.6) | <0.001 |
| mg/dL | 40 (34-47) | 53 (45-61) | 68 (59-78) | 89 (77-100) | <0.001 |
| Apolipoprotein A1, mg/dL | 122 (115-126) | 146 (139-153) | 172 (166-180) | 205 (197-220) | <0.001 |
| Triglycerides |  |  |  |  |  |
| mmol/L | 1.5 (1-2.1) | 1.5 (1.2.2) | 1.4 (0.9-2.0) | 1.2 (0.9-1.8) | <0.001 |
| mg/dL | 128 (88-185) | 128 (88-189) | 119 (83-178) | 108 (78-159) | <0.001 |

Data is summarized as medians (IQR), or numbers (%). *P* values were obtained from the Pearson’s chi-square or Kruskal–Wallis test. HDL=high-density lipoprotein.
 ^a^Included only former and current smokers.
^b^12 grams=1 unit of alcohol.
^c^Being completely sedentary or doing light physical activity less than 2 hours/week.
^d^<9 years of school attendance.
^e^Annual household income <200 000 DKK (approximately 30 000 USD).

**Table S3.** Baseline characteristics of individuals in the Copenhagen City Heart Study according to HDL cholesterol levels.

|  | **HDL cholesterol, mmol/L (mg/dL)** | | | |  |
| --- | --- | --- | --- | --- | --- |
|  | **<1.0 (<39)**  **n=662** | **1.0-1.49 (39-58)**  **n=3523** | **1.5-1.99 (58-77)**  **n=3316** | **≥2.0 (≥77)**  **n=1886** | ***P* value** |
| Age, years | 61 (50-71) | 61 (48-71) | 61 (47-71) | 64 (53-72) | <0.001 |
| Men, no. (%) | 503 (76) | 2068 (59) | 1153 (35) | 397 (21) | <0.001 |
| Body mass index, kg/m^2^ | 27.8 (25.1-30.9) | 26.0 (23.5-28.9) | 24.3 (22.0-27.1) | 23.2 (21.2-25.7) | <0.001 |
| Current smokers, no. (%) | 372 (56) | 1804 (51) | 1595 (48) | 837 (44) | <0.001 |
| Cumulative tobacco consumption, pack-years^a^ | 32 (20-45) | 29 (15-43) | 25 (12-40) | 24 (12-40) | <0.001 |
| Alcohol consumption, units/week^b^ | 4 (0-11) | 5 (0-12) | 5 (1-12) | 7 (2-14) | <0.001 |
| Physical inactivity, no. (%)^c^ | 125 (19) | 461 (13) | 384 (12) | 208 (11) | <0.001 |
| Low education, no. (%)^d^ | 281 (43) | 1315 (38) | 1140 (35) | 599 (32) | <0.001 |
| Low annual household income, no. (%)^e^ | 156 (24) | 771 (22) | 669 (21) | 414 (23) | 0.14 |
| Lipid-lowering therapy, no. (%) | 15 (2.3) | 36 (1.0) | 32 (1.0) | 8 (0.4) | 0.001 |
| High-sensitive C-reactive protein, mg/dL | 2.4 (1.5-4.8) | 1.9 (1.3-3.5) | 1.7 (1.2-2.8) | 1.5 (1.2-2.4) | <0.001 |
| Any prevalent chronic disease, no. (%) | 145 (22) | 418 (12) | 277 (8.4) | 143 (7.6) | <0.001 |
| Ischemic heart disease, no. (%) | 75 (11) | 205 (5.8) | 147 (4.4) | 55 (2.9) | <0.001 |
| Diabetes, no. (%) | 81 (12) | 209 (6.0) | 100 (3.0) | 39 (2.1) | <0.001 |
| Chronic obstructive pulmonary disease, no. (%) | 13 (2.0) | 47 (1.3) | 53 (1.6) | 58 (3.1) | <0.001 |
| HDL cholesterol |  |  |  |  |  |
| mmol/L | 0.9 (0.8-0.9) | 1.2 (1.1-1.3) | 1.7 (1.6-1.8) | 2.2 (2.1-2.5) | <0.001 |
| mg/dL | 35 (31-35) | 46 (42-50) | 66 (62-69) | 85 (81-97) | <0.001 |
| Apolipoprotein A1, mg/dL | 100 (92-107) | 123 (113-133) | 147 (137-158) | 178 (165-193) | <0.001 |
| Triglycerides |  |  |  |  |  |
| mmol/L | 2.8 (1.9-4.2) | 1.9 (1.4-2.6( | 1.4 (1.0-1.9) | 1.2 (0.9-1.5) | <0.001 |
| mg/dL | 249 (166-372) | 164 (119-231) | 123 (91-166) | 101 (77-133) | <0.001 |

Data is summarized as medians (IQR), or numbers (%). *P* values were obtained from the Pearson’s chi-square or Kruskal–Wallis test. HDL=high-density lipoprotein.
 ^a^Included only former and current smokers.
^b^12 grams=1 unit of alcohol.
^c^Being completely sedentary or doing light physical activity less than 2 hours/week.
^d^<8 years of school attendance.
^e^Annual household income <100 000 DKK (approximately 15 000 USD).

**Table S4.** Baseline characteristics of individuals in the Copenhagen City Heart Study according to apolipoprotein A1 levels.

|  | **Apolipoproteinn A1, mg/dL** | | | |  |
| --- | --- | --- | --- | --- | --- |
|  | **<130**  **n=3393** | **130-159**  **n=3604** | **160-189**  **n=1781** | **≥190**  **n=577** | ***P* value** |
| Age, years | 59 (45-70) | 61 (48-71) | 64 (53-72) | 66 (56-72) | <0.001 |
| Men, no. (%) | 2200 (65) | 1413 (39) | 425 (24) | 71 (12) | <0.001 |
| Body mass index, kg/m^2^ | 25.9 (23.3-28.9) | 24.8 (22.4-27.8) | 24.0 (21.7-26.8) | 23.1 (21.1-25.6) | <0.001 |
| Current smokers, no. (%) | 1820 (54) | 1703 (47) | 816 (46) | 252 (44) | <0.001 |
| Cumulative tobacco consumption, pack-years^a^ | 30 (15-43) | 25 (13-40) | 25 (12-40) | 25 (12-39) | <0.001 |
| Alcohol consumption, units/week^b^ | 5 (0-11) | 5 (1-13) | 6 (2-14) | 8 (2-15) | <0.001 |
| Physical inactivity, no. (%)^c^ | 524 (16) | 388 (11) | 199 (11) | 65 (11) | <0.001 |
| Low education, no. (%)^d^ | 1232 (37) | 1266 (35) | 631 (36) | 197 (45) | 0.65 |
| Low annual household income, no. (%)^e^ | 740 (22) | 740 (21) | 395 (23) | 129 (24) | 0.34 |
| Lipid-lowering therapy, no. (%) | 47 (1.4) | 25 (0.7) | 16 (0.9) | 3 (0.5) | 0.02 |
| High-sensitive C-reactive protein, mg/dL | 2.0 (1.3-3.7) | 1.7 (1.2-2.8) | 1.6 (1.2-2.5) | 1.7 (1.2-2.6) | <0.001 |
| Any prevalent chronic disease, no. (%) | 458 (14) | 334 (9.3) | 137 (7.7) | 50 (8.7) | <0.001 |
| Ischemic heart disease, no. (%) | 231 (6.8) | 165 (4.6) | 64 (3.6) | 19 (3.3) | <0.001 |
| Diabetes, no. (%) | 230 (6.8) | 140 (3.9) | 45 (2.5) | 13 (2.3) | <0.001 |
| Chronic obstructive pulmonary disease, no. (%) | 52 (1.5) | 57 (1.6) | 41 (2.3) | 21 (3.6) | 0.001 |
| HDL cholesterol |  |  |  |  |  |
| mmol/L | 1.2 (1.0-1.3) | 1.6 (1.4-1.7) | 2.0 (1.8-2.2) | 2.6 (2.3-2.9) | <0.001 |
| mg/dL | 46 (39-50) | 62 (54-66) | 77 (69-85) | 100 (89-112) | <0.001 |
| Apolipoprotein A1, mg/dL | 116 (106-123) | 143 (136-151) | 171 (165-178) | 202 (195-214) | <0.001 |
| Triglycerides |  |  |  |  |  |
| mmol/L | 1.7 (1.2-2.6) | 1.5 (1.1-2.2) | 1.4 (1.0-1.9) | 1.3 (1.0-1.7) | <0.001 |
| mg/dL | 153 (106-230) | 134 (95-189) | 119 (95-189) | 114 (86-153) | <0.001 |

Data is summarized as medians (IQR), or numbers (%). *P* values were obtained from the Pearson’s chi-square or Kruskal–Wallis test. HDL=high-density lipoprotein.
^a^Included only former and current smokers.
^b^12 grams=1 unit of alcohol.
^c^Being completely sedentary or doing light physical activity less than 2 hours/week.
^d^<8 years of school attendance.
^e^Annual household income <100 000 DKK (approximately 15 000 USD).

**
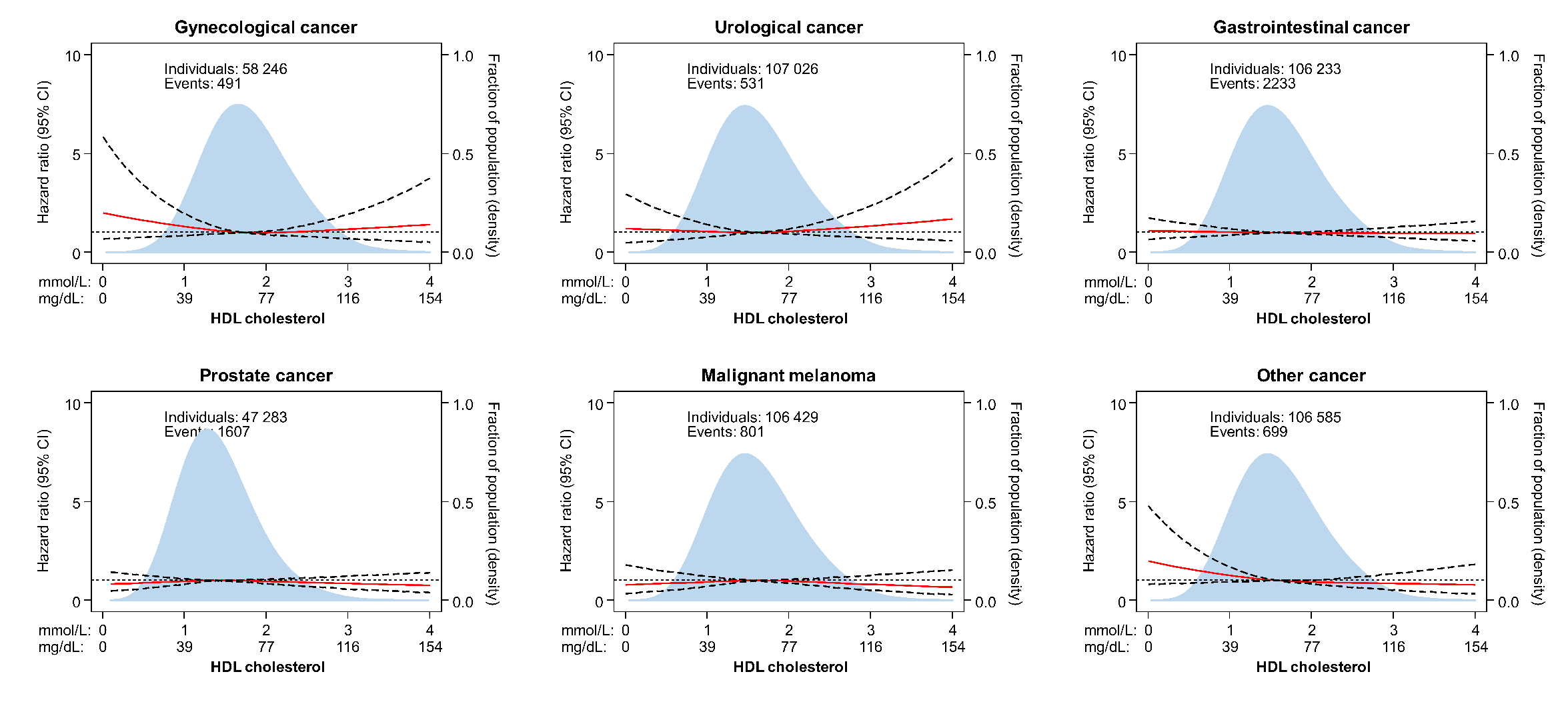

Figure S1. Association of HDL cholesterol levels with risk of gynecological, urological, gastrointestinal, prostate, malignant melanoma, and other cancers in individuals from the Copenhagen General Population Study.**
Hazard ratios and 95% confidence intervals (CIs) were obtained from Cox proportional hazards regression with restricted cubic splines multivariable adjusted for age, sex, body mass index, smoking status, cumulative tobacco consumption, alcohol intake, leisure-time physical activity, education, income, plasma triglycerides, lipid-lowering therapy, C-reactive protein, and baseline chronic disease (ischemic heart disease, chronic obstructive pulmonary disease, and diabetes). The median value of HDL cholesterol was chosen as reference. The red line represents the hazard ratio and the dotted lines 95% CIs. Areas of light blue represent the distribution of levels of HDL cholesterol. Numbers vary slightly due to exclusion of individuals with baseline cancer relevant for the specific cancer form. Gynecological cancer included: cervix uteri, corpus uteri, and ovaries. Urological cancer included: kidney, bladder, and excretory urinary tract. Gastrointestinal cancer included: oral cavity and pharynx, esophagus, stomach, colon/rectum/anus, liver and biliary tract, and pancreas. HDL=high-density lipoprotein.

**
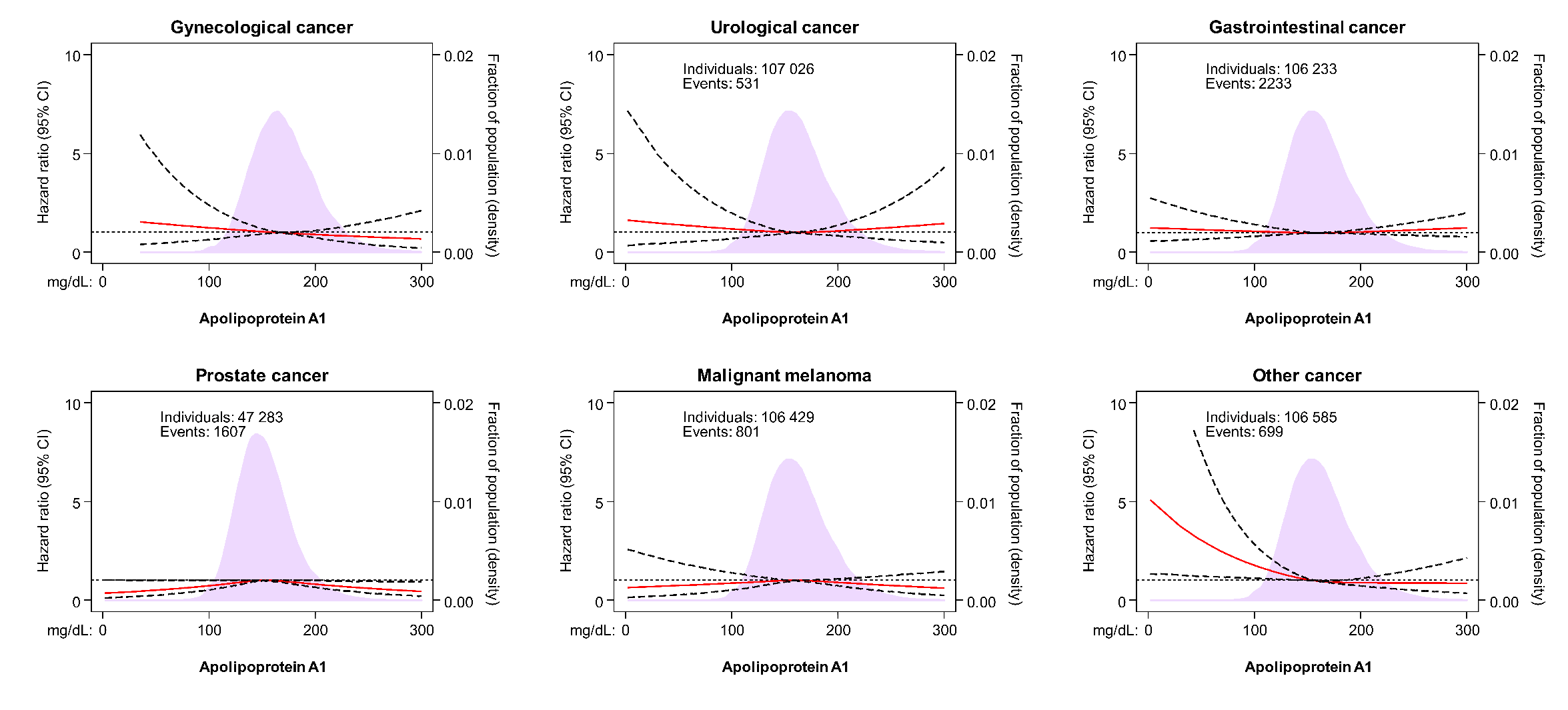

Figure S2. Association of apolipoprotein A1 levels with risk of gynecological, urological, gastrointestinal, prostate, malignant melanoma, and other cancers in individuals from the Copenhagen General Population Study.**
Hazard ratios and 95% confidence intervals (CIs) were obtained from Cox proportional hazards regression with restricted cubic splines multivariable adjusted for age, sex, body mass index, smoking status, cumulative tobacco consumption, alcohol intake, leisure-time physical activity, education, income, plasma triglycerides, lipid-lowering therapy, C-reactive protein, and baseline chronic disease (ischemic heart disease, chronic obstructive pulmonary disease, and diabetes). The median value of apolipoprotein A1 was chosen as reference. The red line represents the hazard ratio and the dotted lines 95% CIs. Areas of purple represent the distribution of levels of apolipoprotein A1. Numbers vary slightly due to exclusion of individuals with baseline cancer relevant for the specific cancer form. Gynecological cancer included: cervix uteri, corpus uteri, and ovaries. Urological cancer included: kidney, bladder, and excretory urinary tract. Gastrointestinal cancer included: oral cavity and pharynx, esophagus, stomach, colon/rectum/anus, liver and biliary tract, and pancreas.

**
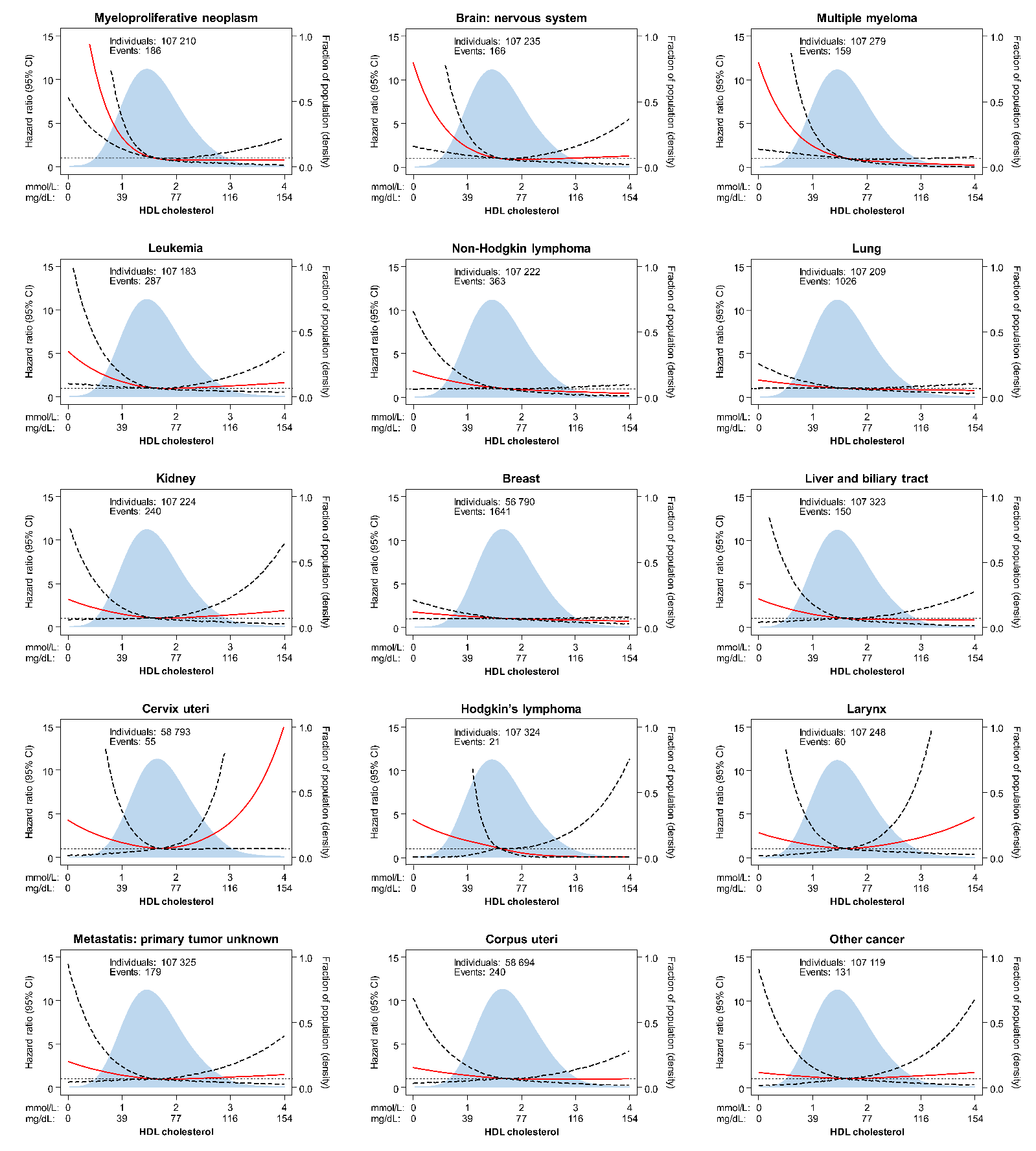

Figure S3a. Association of HDL cholesterol levels with risk of 27 specific cancer types in individuals from the Copenhagen General Population Study.**
Hazard ratios and 95% confidence intervals (CIs) were obtained from Cox proportional hazards regression with restricted cubic splines multivariable adjusted for age, body mass index, smoking status, cumulative tobacco consumption, alcohol intake, leisure-time physical activity, education, income, plasma triglycerides, lipid-lowering therapy, C-reactive protein, and baseline chronic disease (ischemic heart disease, chronic obstructive pulmonary disease, and diabetes). The median value of HDL cholesterol was chosen as reference. The red line represents the hazard ratio and the dotted lines 95% CIs. Areas of light blue represent the distribution of levels of HDL cholesterol. Numbers vary slightly due to exclusion of individuals with baseline cancer relevant for the specific cancer type. HDL=high-density lipoprotein.

**
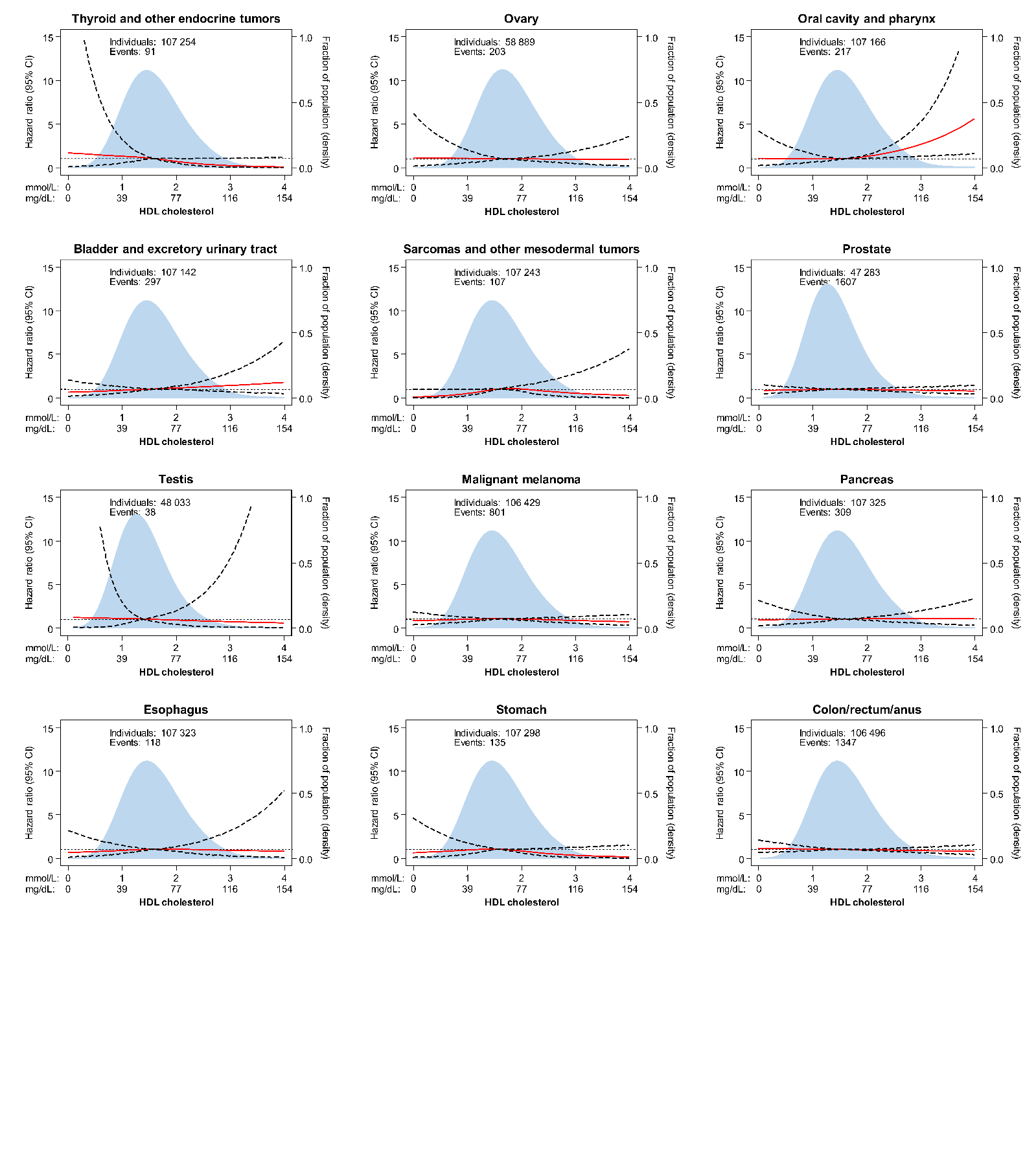
Figure S3b. Association of HDL cholesterol levels with risk of 27 specific cancer types in individuals from the Copenhagen General Population Study.**
Hazard ratios and 95% confidence intervals (CIs) were obtained from Cox proportional hazards regression with restricted cubic splines multivariable adjusted for age, body mass index, smoking status, cumulative tobacco consumption, alcohol intake, leisure-time physical activity, education, income, plasma triglycerides, lipid-lowering therapy, C-reactive protein, and baseline chronic disease (ischemic heart disease, chronic obstructive pulmonary disease, and diabetes). The median value of HDL cholesterol was chosen as reference. The red line represents the hazard ratio and the dotted lines 95% CIs. Areas of light blue represent the distribution of levels of HDL cholesterol. Numbers vary slightly due to exclusion of individuals with baseline cancer relevant for the specific cancer type. HDL=high-density lipoprotein.

**
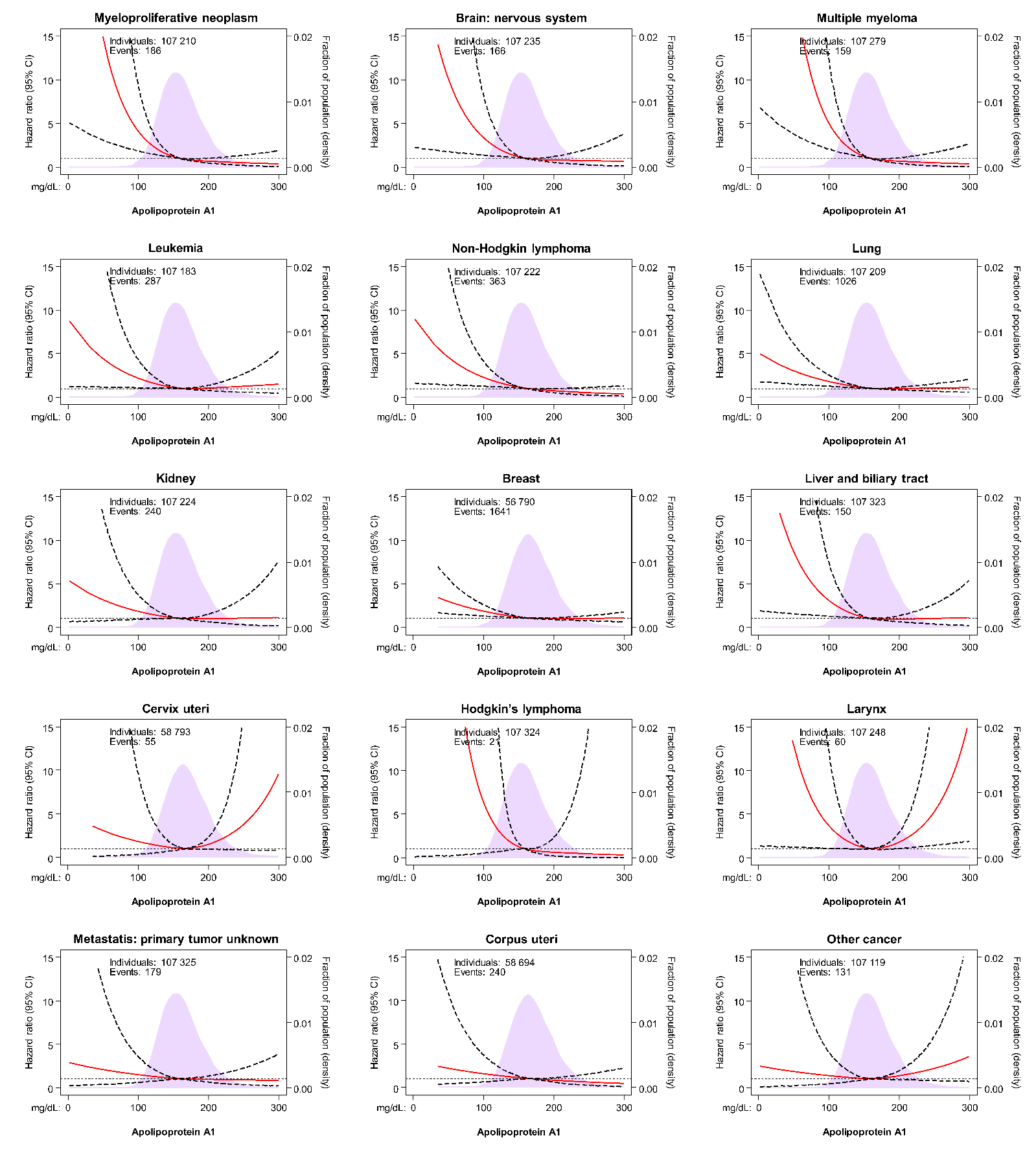

Figure S4a. Association of apolipoprotein A1 levels with risk of 27 specific cancer types in individuals from the Copenhagen General Population Study.**
Hazard ratios and 95% confidence intervals (CIs) were obtained from Cox proportional hazards regression with restricted cubic splines multivariable adjusted for age, body mass index, smoking status, cumulative tobacco consumption, alcohol intake, leisure-time physical activity, education, income, plasma triglycerides, lipid-lowering therapy, C-reactive protein, and baseline chronic disease (ischemic heart disease, chronic obstructive pulmonary disease, and diabetes). The median value of apolipoprotein A1 was chosen as reference. The red line represents the hazard ratio and the dotted lines 95% CIs. Areas of purple represent the distribution of levels of apolipoprotein A1. Numbers vary slightly due to exclusion of individuals with baseline cancer relevant for the specific cancer type.

**
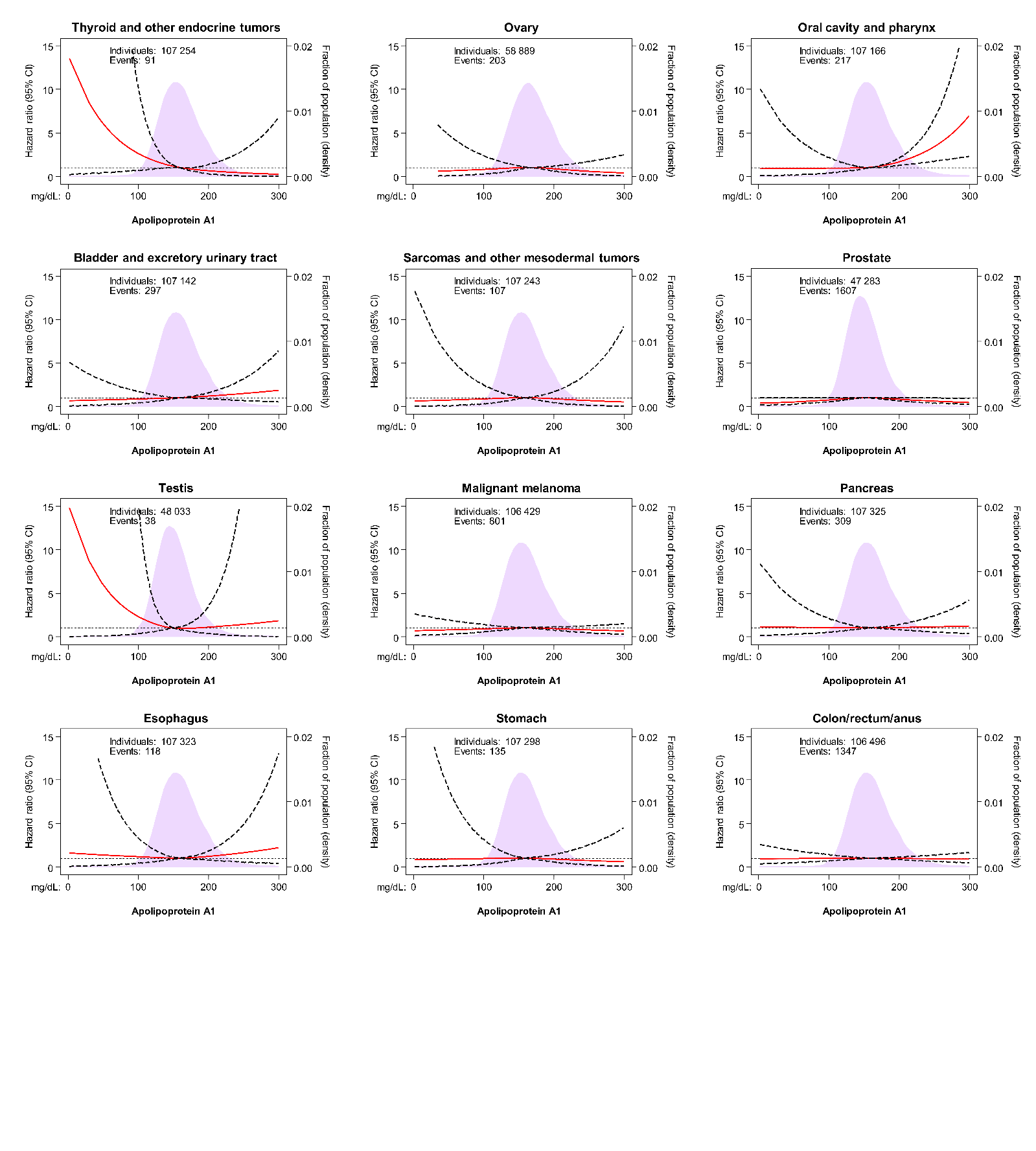
Figure S4b. Association of apolipoprotein A1 levels with risk of 27 specific cancer types in individuals from the Copenhagen General Population Study.**
Hazard ratios and 95% confidence intervals (CIs) were obtained from Cox proportional hazards regression with restricted cubic splines multivariable adjusted for age, body mass index, smoking status, cumulative tobacco consumption, alcohol intake, leisure-time physical activity, education, income, plasma triglycerides, lipid-lowering therapy, C-reactive protein, and baseline chronic disease (ischemic heart disease, chronic obstructive pulmonary disease, and diabetes). The median value of apolipoprotein A1 was chosen as reference. The red line represents the hazard ratio and the dotted lines 95% CIs. Areas of purple represent the distribution of levels of apolipoprotein A1. Numbers vary slightly due to exclusion of individuals with baseline cancer relevant for the specific cancer type.

**
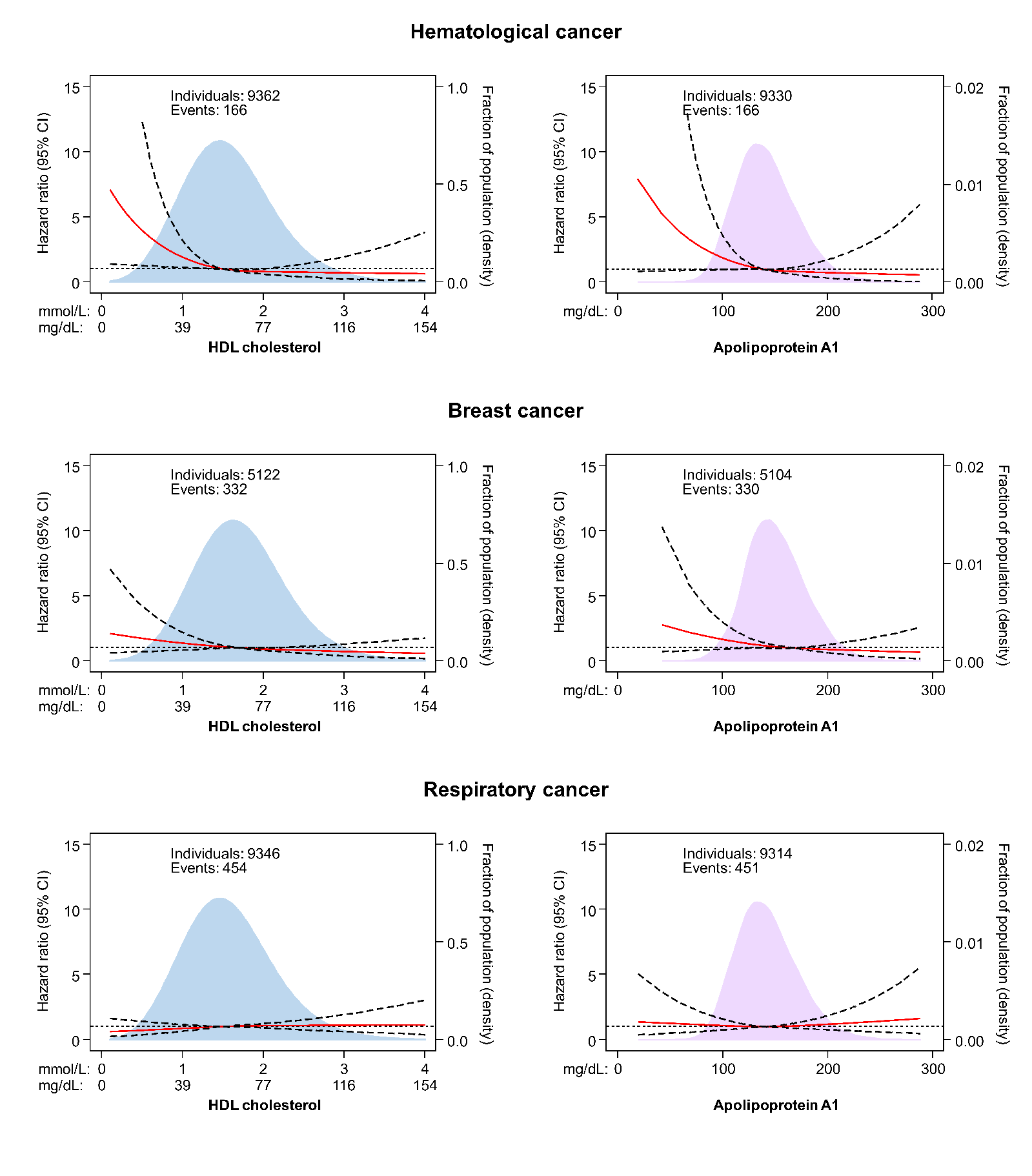
Figure S5. Association of HDL cholesterol and apolipoprotein A1 levels with risk of hematological, breast, and respiratory cancers in individuals from the Copenhagen City Heart Study.**Hazard ratios and 95% confidence intervals (CIs) were obtained from Cox proportional hazards regression with restricted cubic splines multivariable adjusted for age, sex, body mass index, smoking status, cumulative tobacco consumption, alcohol intake, leisure-time physical activity, education, income, plasma triglycerides, lipid-lowering therapy, C-reactive protein, and baseline chronic disease (ischemic heart disease, chronic obstructive pulmonary disease, and diabetes). The median values of HDL cholesterol and apolipoprotein A1 were chosen as reference. The red line represents the hazard ratio and the dotted lines 95% CIs. Areas of light blue and purple represent the distribution of levels of HDL cholesterol and apolipoprotein A1, respectively. Numbers vary slightly due to exclusion of individuals with baseline cancer relevant for the specific cancer form. Hematological cancer included: non-Hodgkin lymphoma, Hodgkin’s lymphoma, multiple myeloma, leukemia, and myeloproliferative neoplasm. Respiratory cancer included: larynx and lung. HDL=high-density lipoprotein.

**
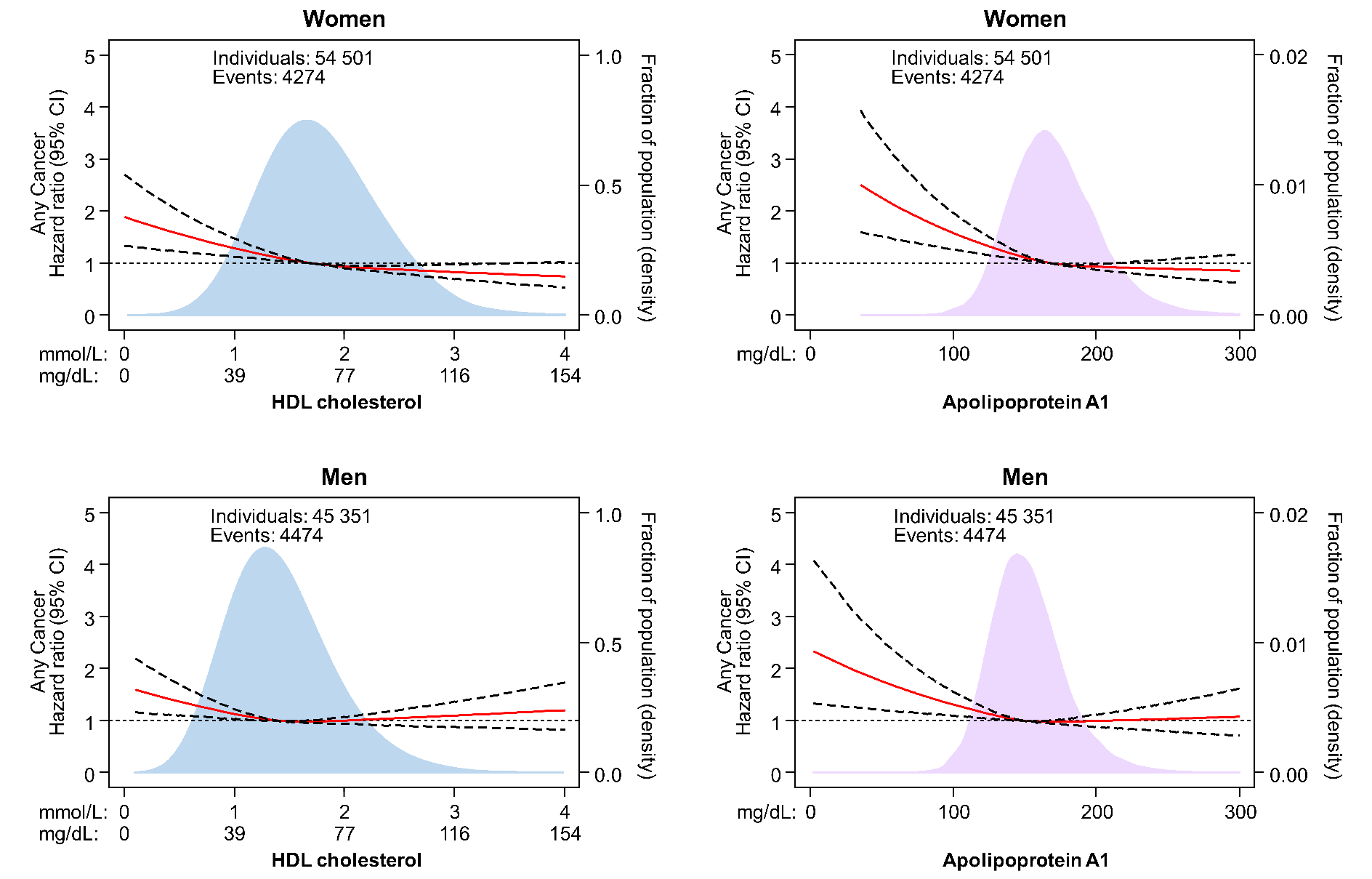

Figure S6. Association of HDL cholesterol and apolipoprotein A1 levels with risk of any cancer in individuals from the Copenhagen General Population Study according to sex.**
Hazard ratios and 95% confidence intervals (CIs) were obtained from Cox proportional hazards regression with restricted cubic splines. Multivariable adjustment included age, body mass index, smoking status, cumulative tobacco consumption, alcohol intake, leisure-time physical activity, education, income, plasma triglycerides, lipid-lowering therapy, C-reactive protein, and baseline chronic disease (ischemic heart disease, chronic obstructive pulmonary disease, and diabetes). The median values of HDL cholesterol and apolipoprotein A1 were chosen as reference. The red line represents the hazard ratio and the dotted lines 95% CIs. Areas of light blue and purple represent the distribution of levels of HDL cholesterol and apolipoprotein A1, respectively. HDL=high-density lipoprotein.

**
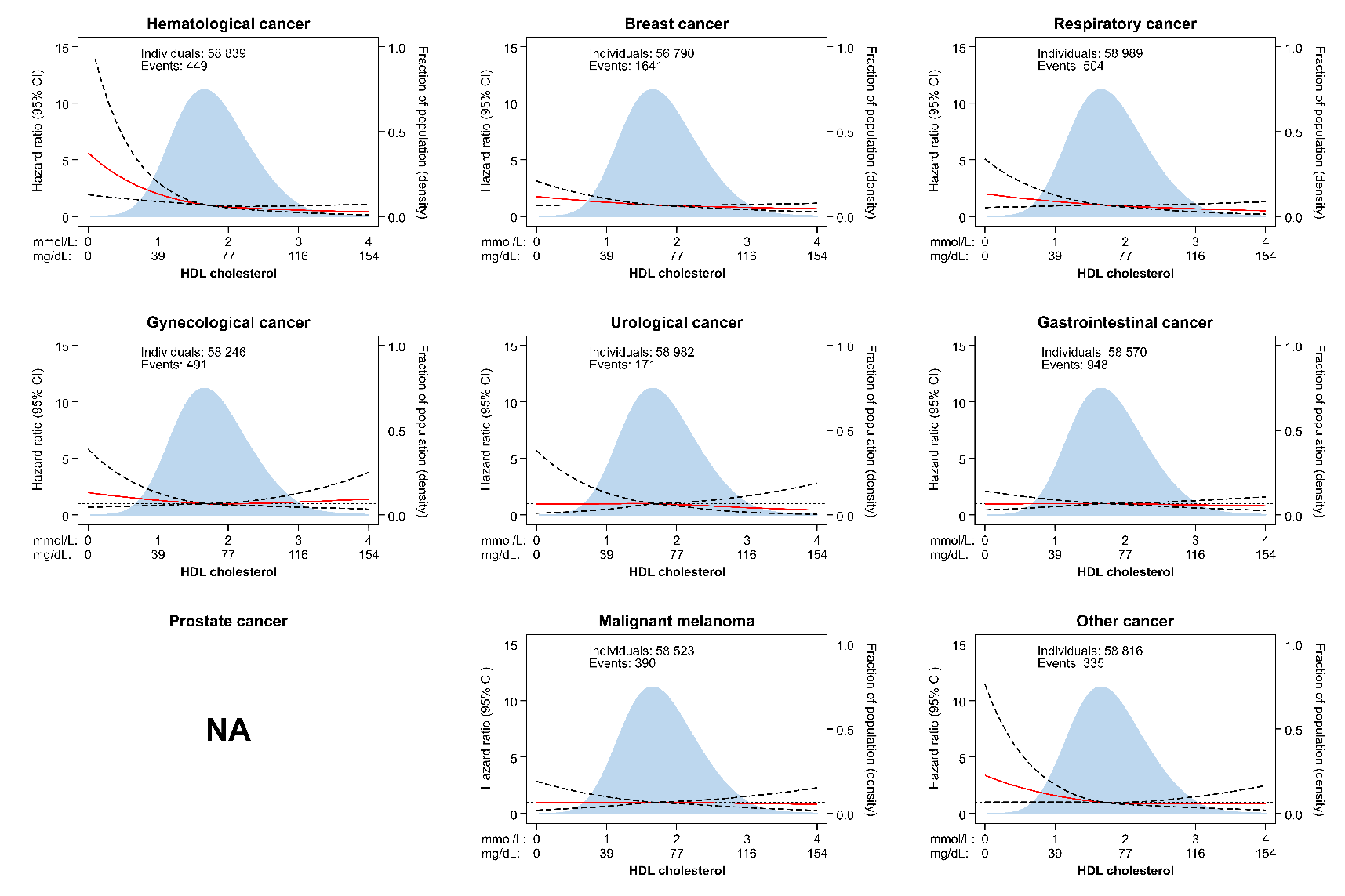

Figure S7. Association of HDL cholesterol levels with risk of eight major cancer forms in women from the Copenhagen General Population Study.**
Hazard ratios and 95% confidence intervals (CIs) were obtained from Cox proportional hazards regression with restricted cubic splines multivariable adjusted for age, body mass index, smoking status, cumulative tobacco consumption, alcohol intake, leisure-time physical activity, education, income, plasma triglycerides, lipid-lowering therapy, C-reactive protein, and baseline chronic disease (ischemic heart disease, chronic obstructive pulmonary disease, and diabetes). The median value of HDL cholesterol was chosen as reference. The red line represents the hazard ratio and the dotted lines 95% CIs. Areas of light blue represent the distribution of levels of HDL cholesterol. Numbers vary slightly due to exclusion of individuals with baseline cancer relevant for the specific cancer form. Hematological cancer included: non-Hodgkin lymphoma, Hodgkin’s lymphoma, multiple myeloma, leukemia, and myeloproliferative neoplasm. Respiratory cancer included: larynx and lung. Gynecological cancer included: cervix uteri, corpus uteri, and ovaries. Urological cancer included: kidney, bladder, and excretory urinary tract. Gastrointestinal cancer included: oral cavity and pharynx, esophagus, stomach, colon/rectum/anus, liver and biliary tract, and pancreas. HDL=high-density lipoprotein.


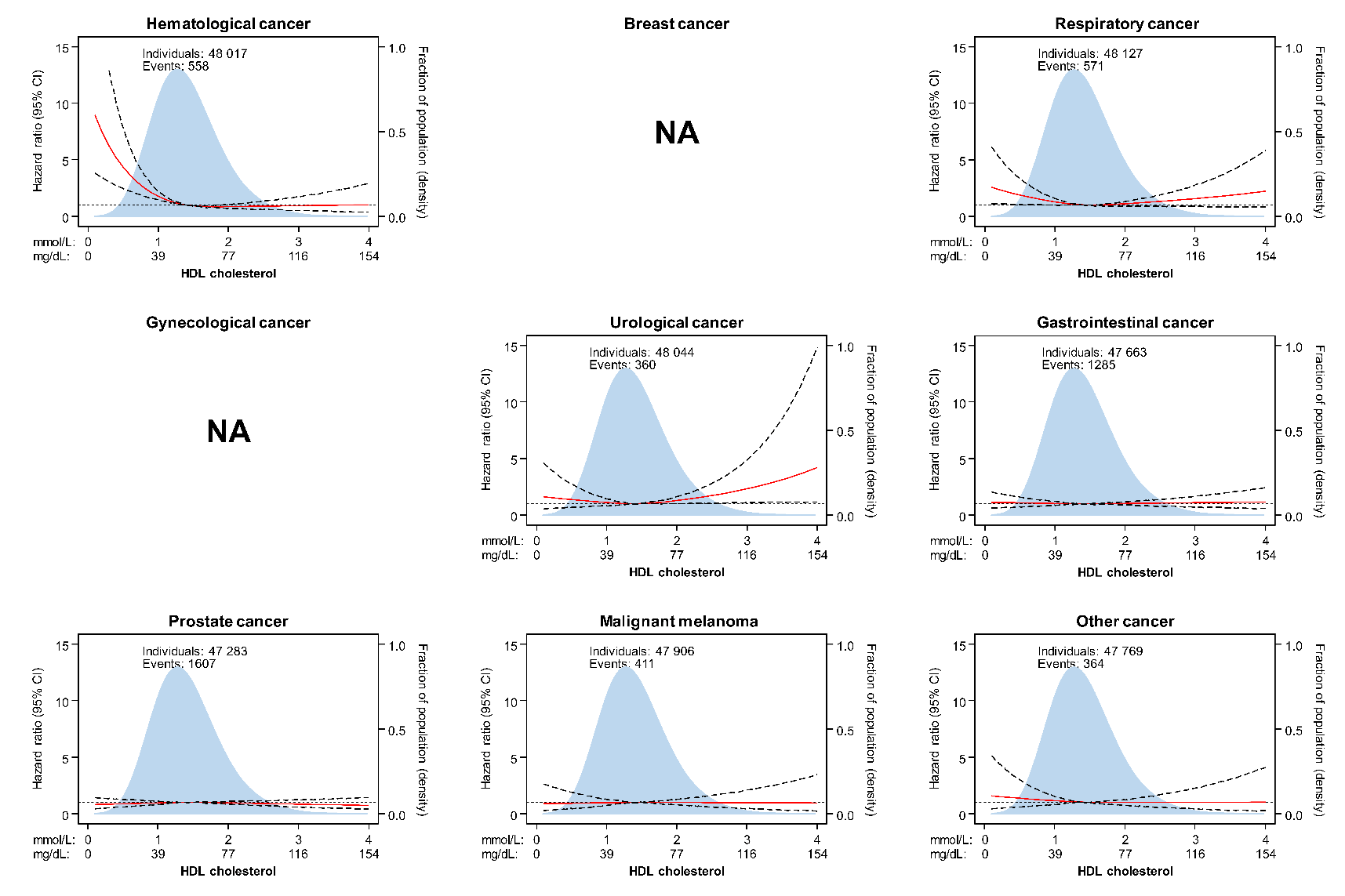


**Figure S8. Association of HDL cholesterol levels with risk of seven major cancer forms in men from the Copenhagen General Population Study.**
Hazard ratios and 95% confidence intervals (CIs) were obtained from Cox proportional hazards regression with restricted cubic splines multivariable adjusted for age, body mass index, smoking status, cumulative tobacco consumption, alcohol intake, leisure-time physical activity, education, income, plasma triglycerides, lipid-lowering therapy, C-reactive protein, and baseline chronic disease (ischemic heart disease, chronic obstructive pulmonary disease, and diabetes). The median value of HDL cholesterol was chosen as reference. The red line represents the hazard ratio and the dotted lines 95% CIs. Areas of light blue represent the distribution of levels of HDL cholesterol. Numbers vary slightly due to exclusion of individuals with baseline cancer relevant for the specific cancer form. Hematological cancer included: non-Hodgkin lymphoma, Hodgkin’s lymphoma, multiple myeloma, leukemia, and myeloproliferative neoplasm. Respiratory cancer included: larynx and lung. Gynecological cancer included: cervix uteri, corpus uteri, and ovaries. Urological cancer included: kidney, bladder, and excretory urinary tract. Gastrointestinal cancer included: oral cavity and pharynx, esophagus, stomach, colon/rectum/anus, liver and biliary tract, and pancreas. HDL=high-density lipoprotein.

**
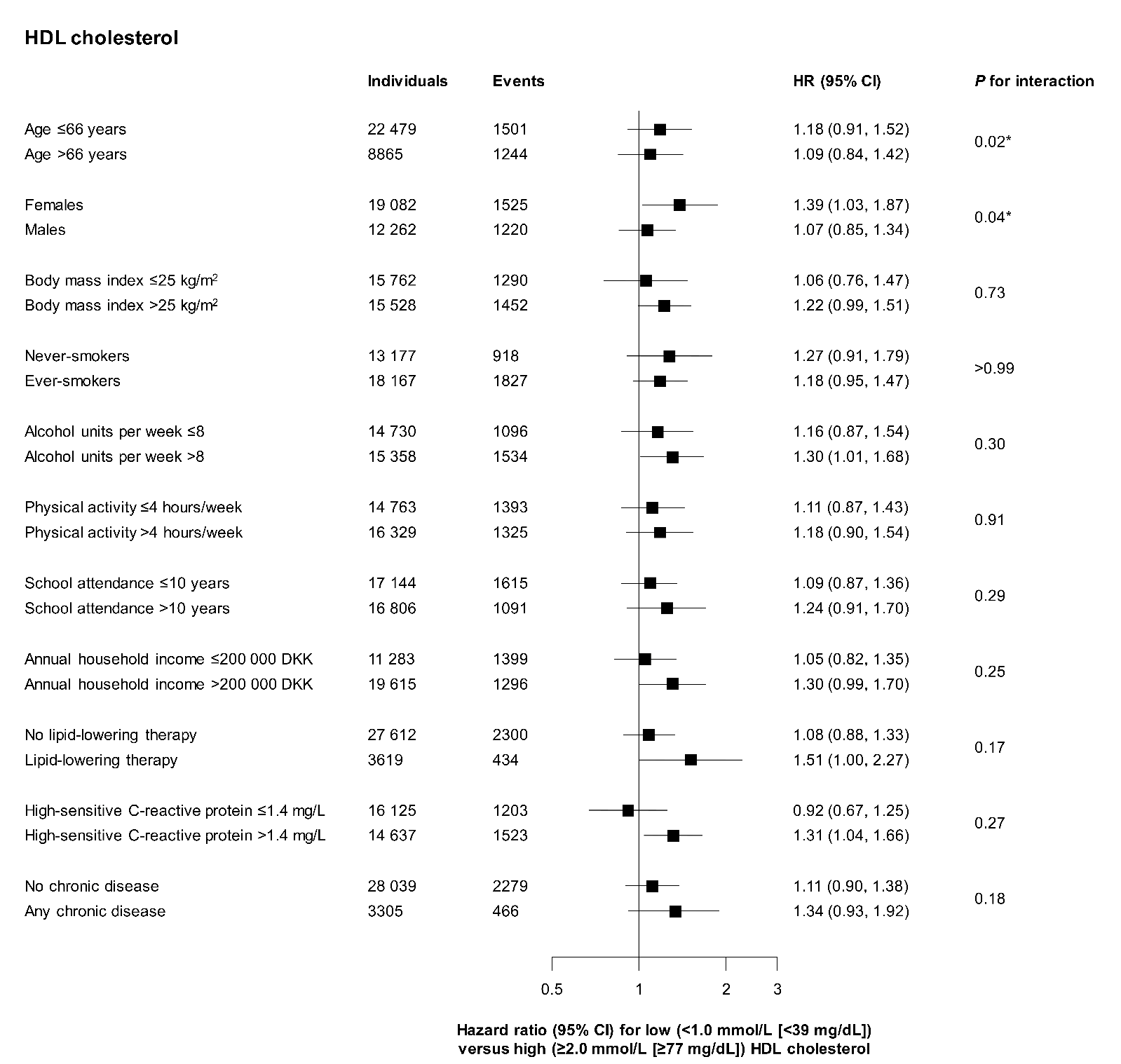


Figure S9. Association of low versus high HDL cholesterol levels with risk of any cancer in individuals from the Copenhagen General Population Study according to covariates.**Hazard ratios and 95% confidence intervals (CIs) were obtained from Cox proportional hazards for individuals with low (<1.0 mmol/L [<39 mg/dL]) versus high (≥2.0 mmol/L [≥77 mg/dL]) HDL cholesterol, multivariable adjusted for age, body mass index, smoking status, cumulative tobacco consumption, alcohol intake, leisure-time physical activity, education, income, plasma triglycerides, lipid-lowering therapy, C-reactive protein, and baseline chronic disease (ischemic heart disease, chronic obstructive pulmonary disease, and diabetes). *P* for interaction was obtained from Wald’s test for interaction. Age=66 years was the median age for cancer cases at baseline. Chronic diseases included ischemic heart disease, diabetes, and chronic obstructive pulmonary disease. HDL=high-density lipoprotein. *****When *P* for interaction is adjusted for 11 individual interaction analyses according to the Bonferroni method, *P*=0.05 is equivalent to *P*=0.05/11=0.005.

**
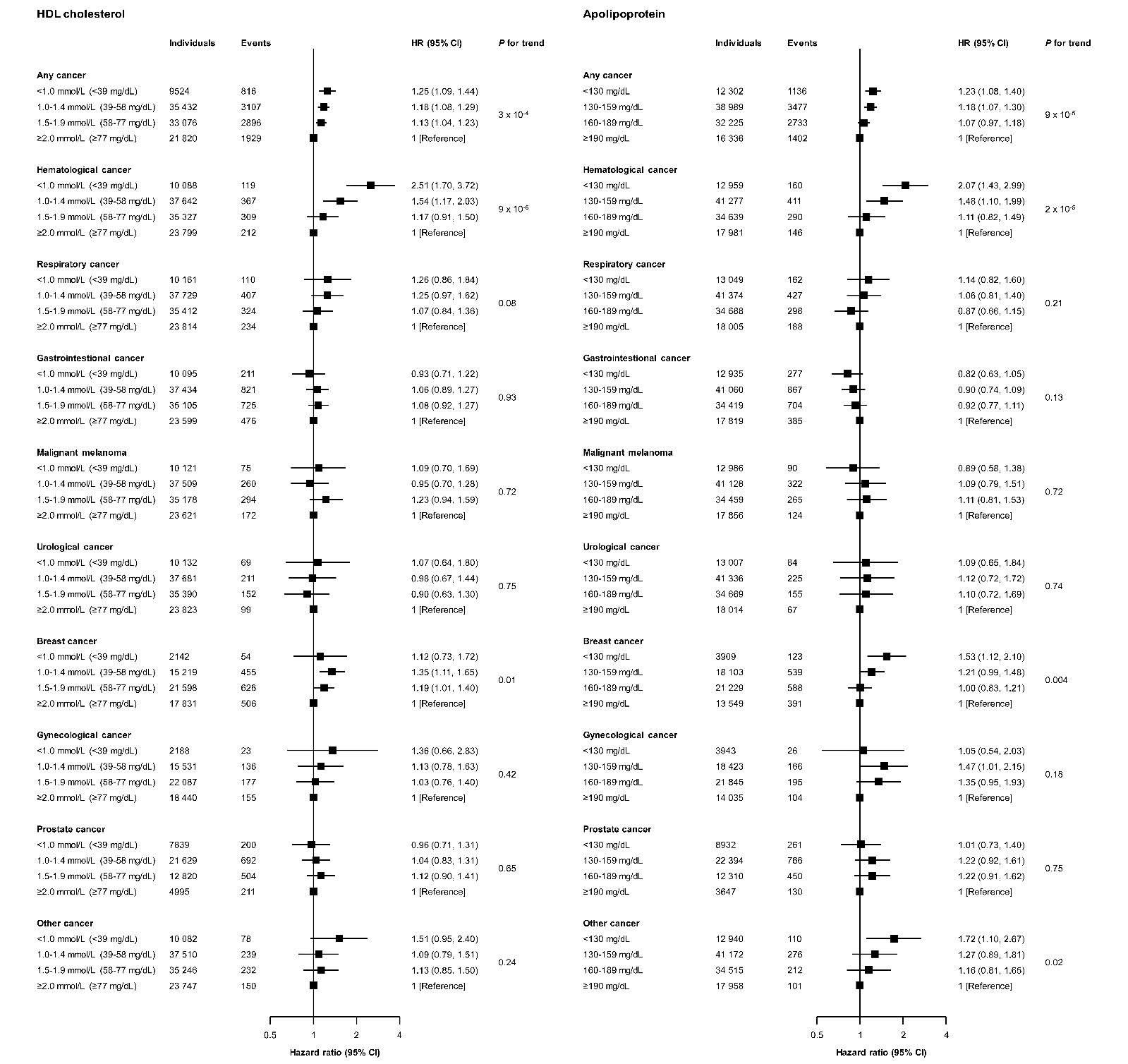

Figure S10. Association of HDL cholesterol and apolipoprotein A1 levels with risk of any cancer and nine major cancer forms in individuals from the Copenhagen General Population Study with all-cause mortality and emigration as competing events.**Subdistribution hazard ratios and 95% confidence intervals (CIs) were obtained from competing-risk analysis according to Fine & Gray with death and emigration as competing events, multivariable adjusted for age, body mass index, smoking status, cumulative tobacco consumption, alcohol intake, leisure-time physical activity, education, income, plasma triglycerides, lipid-lowering therapy, C-reactive protein, and baseline chronic disease (ischemic heart disease, chronic obstructive pulmonary disease, and diabetes). *P* for trend was obtained from Wald’s test. Numbers vary slightly due to exclusion of individuals with baseline cancer relevant for the specific cancer form. The sum of the nine major cancer forms exceeds the number of any cancer, as some individuals developed more than one specific cancer form. Hematological cancer included: non-Hodgkin lymphoma, Hodgkin’s lymphoma, multiple myeloma, leukemia, and myeloproliferative neoplasm. Respiratory cancer included: larynx and lung. Gynecological cancer included: cervix uteri, corpus uteri, and ovaries. Urological cancer included: kidney, bladder, and excretory urinary tract. Gastrointestinal cancer included: oral cavity and pharynx, esophagus, stomach, colon/rectum/anus, liver and biliary tract, and pancreas. HDL=high-density lipoprotein.

**
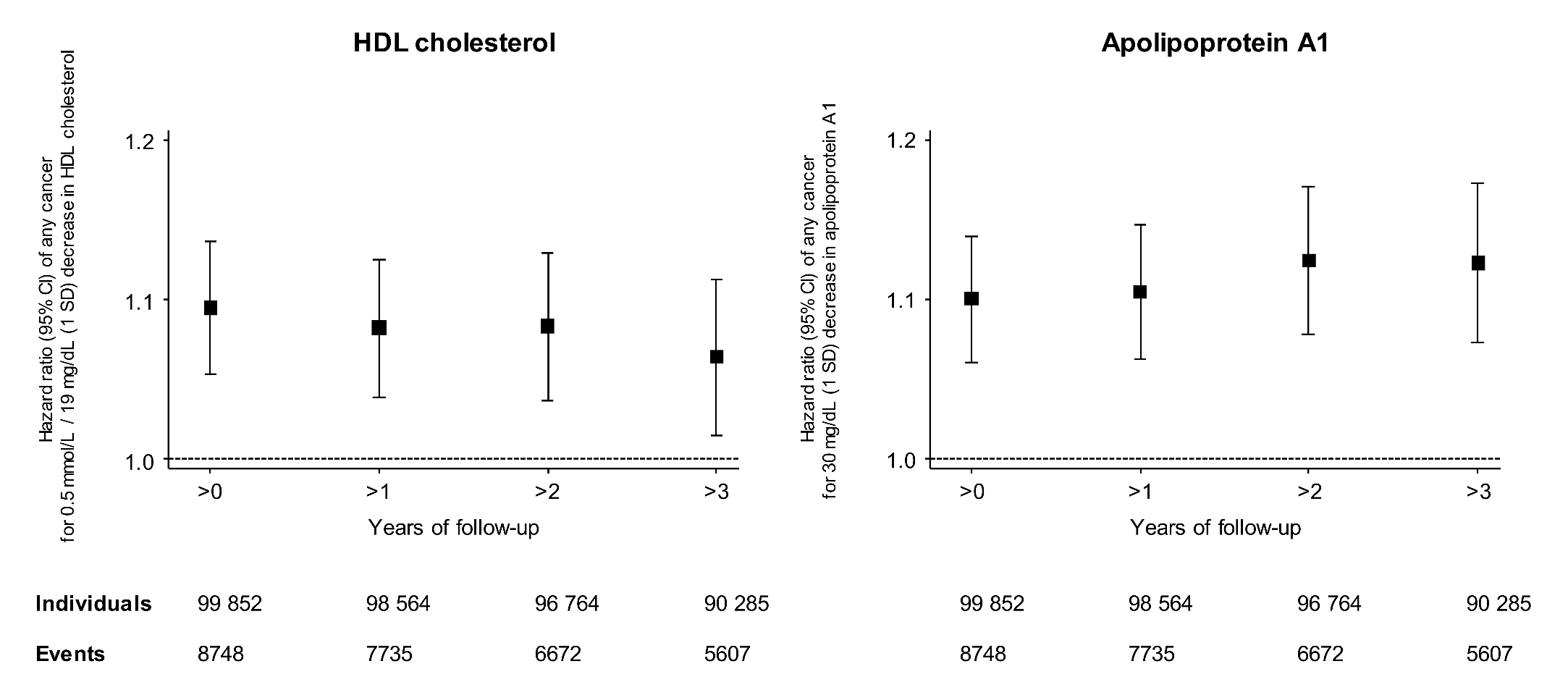

Figure S11. Association of HDL cholesterol and apolipoprotein A1 levels with risk of any cancer in individuals from the Copenhagen General Population Study excluding individuals with 1, 2, and 3 years of follow-up.**Hazard ratios and 95% confidence intervals (CIs) were obtained from Cox proportional hazards regression multivariable adjusted for age, sex, body mass index, smoking status, cumulative tobacco consumption, alcohol intake, leisure-time physical activity, education, income, plasma triglycerides, lipid-lowering therapy, C-reactive protein, and baseline chronic disease (ischemic heart disease, chronic obstructive pulmonary disease, and diabetes). Individuals with <1–3 years of follow-up owing to cancer event, censoring owing to death or emigration, or end of follow-up were excluded as indicated by the respective numbers on the x axis. HDL=high-density lipoprotein.

**
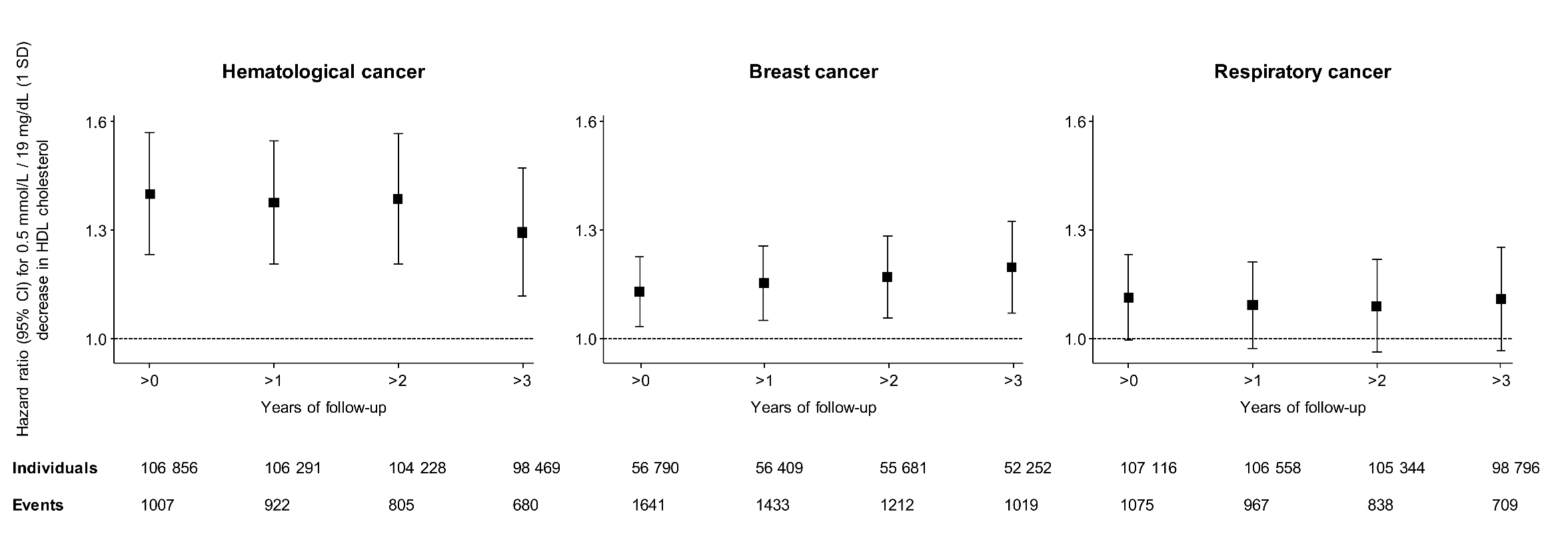

Figure S12. Association of HDL cholesterol levels with risk of hematological, breast, and respiratory cancers in individuals from the Copenhagen General Population Study excluding individuals with 1, 2, and 3 years of follow-up.**Hazard ratios and 95% confidence intervals (CIs) were obtained from Cox proportional hazards regression multivariable adjusted for age, sex, body mass index, smoking status, cumulative tobacco consumption, alcohol intake, leisure-time physical activity, education, income, plasma triglycerides, lipid-lowering therapy, C-reactive protein, and baseline chronic disease (ischemic heart disease, chronic obstructive pulmonary disease, and diabetes). Individuals with <1–3 years of follow-up owing to cancer event, censoring owing to death or emigration, or end of follow-up were excluded as indicated by the respective numbers on the x axis. Numbers vary slightly due to exclusion of individuals with baseline cancer relevant for the specific cancer form. Hematological cancer included: non-Hodgkin lymphoma, Hodgkin’s lymphoma, multiple myeloma, leukemia, and myeloproliferative neoplasm. Respiratory cancer included: larynx and lung. HDL=high-density lipoprotein.

**
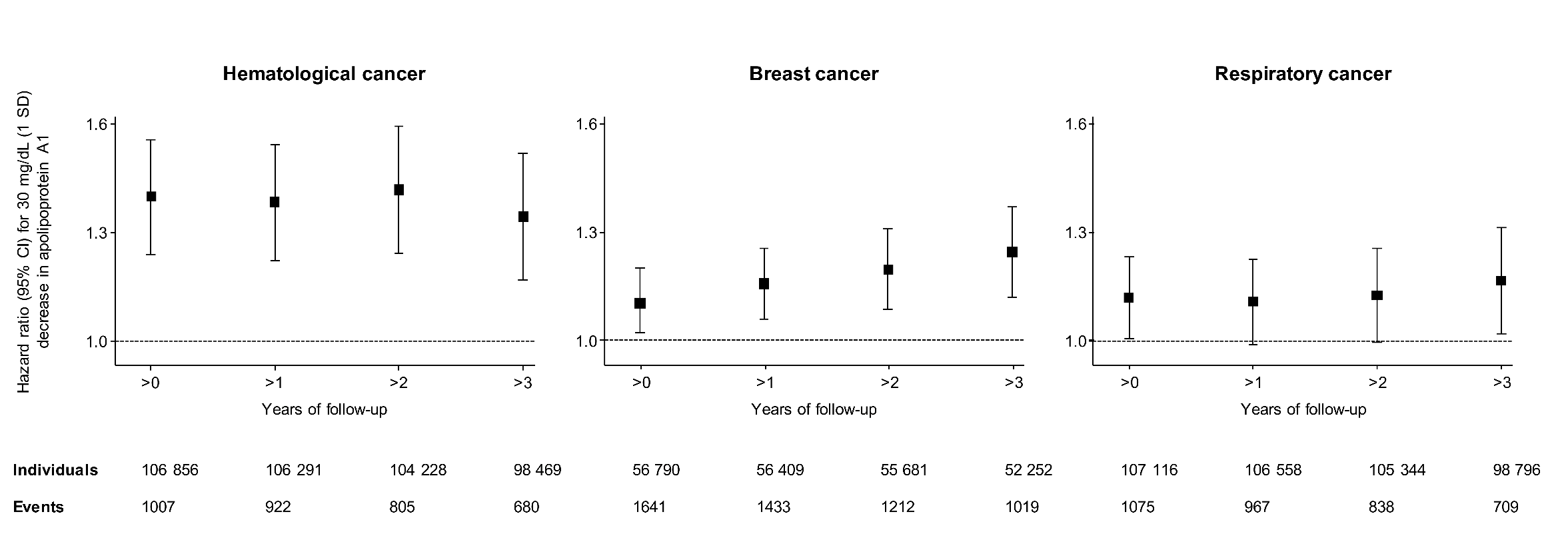

Figure S13. Association of apolipoprotein A1 levels with risk of hematological, breast, and respiratory cancers in individuals from the Copenhagen General Population Study excluding individuals with 1, 2, and 3 years of follow-up.**Hazard ratios and 95% confidence intervals (CIs) were obtained from Cox proportional hazards regression multivariable adjusted for age, sex, body mass index, smoking status, cumulative tobacco consumption, alcohol intake, leisure-time physical activity, education, income, plasma triglycerides, lipid-lowering therapy, C-reactive protein, and baseline chronic disease (ischemic heart disease, chronic obstructive pulmonary disease, and diabetes). Individuals with <1–3 years of follow-up owing to cancer event, censoring owing to death or emigration, or end of follow-up were excluded as indicated by the respective numbers on the x axis. Numbers vary slightly due to exclusion of individuals with baseline cancer relevant for the specific cancer form. Hematological cancer included: non-Hodgkin lymphoma, Hodgkin’s lymphoma, multiple myeloma, leukemia, and myeloproliferative neoplasm. Respiratory cancer included: larynx and lung.

**
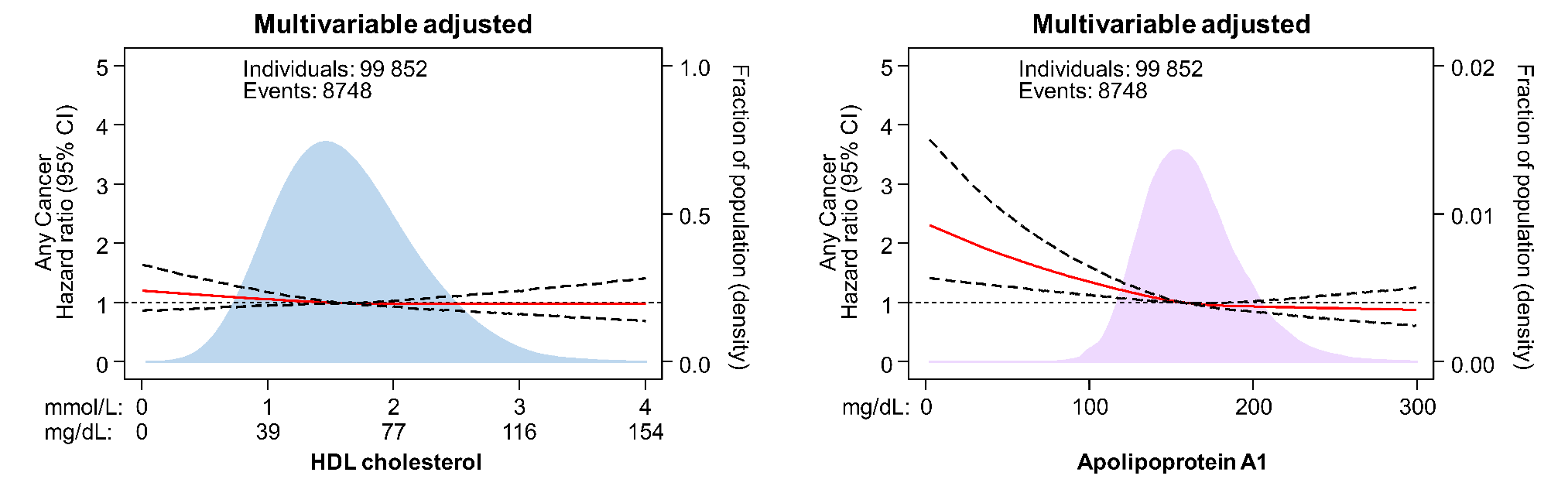

Figure S14. HDL cholesterol and apolipoprotein A1 levels adjusted risk of any cancer in individuals from the Copenhagen General Population Study.**Hazard ratios and 95% confidence intervals (CIs) were obtained from Cox proportional hazards regression with restricted cubic splines including both HDL cholesterol and apolipoprotein A1 as covariates in the model. Multivariable adjustment included age, sex, body mass index, smoking status, cumulative tobacco consumption, alcohol intake, leisure-time physical activity, education, income, plasma triglycerides, lipid-lowering therapy, C-reactive protein, and baseline chronic disease (ischemic heart disease, chronic obstructive pulmonary disease, and diabetes). The median values of HDL cholesterol and apolipoprotein A1 were chosen as reference. The red line represents the hazard ratio and the dotted lines 95% CIs. Areas of light blue and purple represent the distribution of levels of HDL cholesterol and apolipoprotein A1, respectively. HDL=high-density lipoprotein.
